# An Intersectional Analysis of Moral Distress and Intention to Leave Employment Among Long-Term Care Providers in British Columbia

**DOI:** 10.1177/08982643231212981

**Published:** 2023-11-09

**Authors:** Julia Smith, Muhammad Haaris Tiwana, Hasina Samji, Rosemary Morgan, Simran Purewal, Jorge Andres Delgado-Ron

**Affiliations:** 1 Faculty of Health Sciences, 1763 Simon Fraser University, Burnaby, BC, Canada; 2 Department of International Health, Bloomberg School of Public Health, 1466 Johns Hopkins University, Baltimore, MD, USA

**Keywords:** moral distress, long-term care, intersectionality, mitigation, equity

## Abstract

**Objectives:** In this study, we aimed to explore the relationship between intersectional inequities and moral distress among those working in Long-Term Care (LTC) in British Columbia, Canada. **Methods:** This was a cross-sectional and retrospective study. We assessed moral distress, of 1678 respondents, using a modified Moral Distress Scale, and an equivalent distress mitigation score, at the intersections of gender and racial/ethnic identity. Then, we explored which worker attributes were more predictive of intention to leave work. **Results:** We found notable difference in experiences of moral distress across intersecting identities, including high moral distress scores among Indigenous men and women, and white women. Significant differences in mitigation scores were also found by intersectional identities. **Discussion:** Moral distress was the most important predictor of intention to leave work. The differences across racial and gender identity groups suggest the need for tailored interventions to address moral distress among LTC providers.

## Introduction

The concept of moral distress, defined as “a phenomenon in which one knows the ethically right action to take, but is systemically constrained from taking it,” has been increasingly applied to understand the experiences of those working in the Long-Term Care (LTC) sector (Jameton, 2017). The Canadian context provides ample scope through which to study moral distress in LTC (residential care for older adults and others in need of assistance) due to numerous staffing and quality of care challenges. LTC providers in Canada are often subjected to exhausting and unpredictable workloads and inconsistent assignment of work hours, with over 24% working more than one job in 2019 (Duan et al., 2020). Lakusta’s (2018) study found that support workers are retained in the sector fewer than five years. The Canadian Association for Long Term Care (2021) highlighted that there were approximately 35,000 vacant jobs in LTC’s in the latter half of 2021 which is a 350% increase from earlier. Furthermore, it reported that 1 in 4 nurses (24.4%), and 1 in 6 support workers or care aides (16.4%) intended to leave their current job or change jobs within next three years (Statistics Canada, 2022). Those working in LTC report declining emotional well-being, over the past few years, and fear of taking sick leave due to the risk of losing employment (Office of the Seniors Advocate, 2020). Similarly, longitudinal research documents declining mental health among LTC care aides, a trend that the COVID-19 pandemic worsened (Havaei et al., 2022).

Staffing and occupation health challenges reflect the positioning of LTC on the periphery of Canada’s healthcare system. On average, Canada invests 30% less on LTC than other OECD countries, and the sector is fragmented among public, non-profit, and for-profit providers (Estabrooks et al., 2020). Numerous studies have pointed to how cultural ageism is reflected in the lack of both investment and respect for those who staff it (Booi et al., 2021; Pijl-Zieber et al., 2008). Braedley et al.’s (2018) study of occupational health and safety in LTC in Canada finds numerous hazards related to moral distress including work overload, low worker control, disrespect, and discrimination. Greason (2020) looks at ethical reasoning among LTC staff, finding staff typically do not have difficulty determining ethical decisions and/or actions but frequently experience moral distress. Booi et al. (2021) identify a sub-theme related to moral distress in their qualitative research on the experiences of LTC care aides in Canada. Their study notes that cultural attitudes towards care for the elderly influenced experiences of moral distress. Smith et al. (2023) focus on women LTC care aids in their qualitative study of moral distress during the COVID-19 pandemic, documenting how gender norms contribute to moral distress. Both studies highlight specific characteristics of the LTC workforce in Canada that are relevant to understanding the multifaceted nature of moral distress.

In British Columbia (BC), Canada, 77.6% of those working in the health and social assistance sector, which LTC falls within, are women, 45% are immigrants, and 34% are people of color (Statistics Canada, 2020). Research has increasingly documented the discrimination that racialized LTC providers face from both residents and colleagues (Bourgeault et al., 2010; Syed, 2020). Considering the effects of moral distress on personal well-being and career development, it is crucial to understand whether such differences could exacerbate inequities within the health workforce. The consideration of moral distress is essential for fostering a thriving workforce while avoiding its typical negative outcomes that result in the workplace. The provision of ethical, competent, and safe care depends on a vibrant workforce. According to research (Hart, 2005), investing in resources to address ethical concerns is a cost-effective measure that contributes to the overall well-being of the workforce.

Here, we aim to explore the relationship between intersectional inequities and moral distress among those working in LTC in BC, Canada. In doing so, we draw on the work of African American feminists such as Kimberly Crenshaw and Patricia Collins around the concept of intersectionality (Collins, 2015; Crenshaw, 1997). Recognizing that definitions are “a starting point,” as Collins (2015) states, we understand intersectionality as a “framework for understanding how multiple social identities such as race/ethnicity, gender, sexual orientation, SES (Socio-Economic Status), and disability, intersect at the micro level of individual experience to reflect interlocking systems of privilege and oppression (i.e., racism, sexism, heterosexism, classism) at the macro social-structural level” (Bowleg, 2013). While much of the research applying intersectionality is qualitative, critical and/or explorative, quantitative researchers have also begun to engage with intersectional approaches in empirical studies. However, as Bauer et al., 2021 note, little of this work engages with the theoretical underpinnings of intersectionality, instead focusing on differences among select social positions.

Acknowledging the limitations of quantitative methods in deducing the relationship between multiple social positions, we first describe the levels of moral distress at the two-way intersections of gender and racial or ethnic identity among LTC providers, the components of moral distress that stood out in said intersections, and the corresponding mitigation strategies. Secondly, to consider additional intersecting identity factors, we explored which worker attributes (i.e., gender, racialized experiences, profession, work arrangement, migratory information, and professional distress levels) were more predictive of intention to leave work. Finally, recognizing that intersectionality includes a call to not just document but also dismantle interlocking systems of oppression, we included questions around strategies for distress mitigation, to understand how care providers resist the effects of moral distress and how they might be supported in doing so. Including distress mitigation prevents care providers from being positioned as passive actors and it also shifts research from simply documenting moral distress to informing interventions to address it—two prominent critiques of past moral distress research (McCarthy & Gastmans, 2015; Rushton et al., 2016).

## Methods

### Study Design, Setting, and Data Sources

This was an observational, cross-sectional, and retrospective study which was administered through a survey. The study was approved by the Simon Fraser University (SFU) Research Ethics Board (#30001218). We used convenience sampling for the survey. English-speaking physicians, nurses, homecare aids, and long-term care aids were eligible if currently employed in BC at the time of the survey. We recruited through email and social media via work unions, such as the Hospital Employees Union, and occupational organizations, such as SafeCare BC. To encourage participation, a raffle was established with a chance to win one of twenty $100 Visa gift cards. Eligible participants were directed to an online questionnaire hosted on SFU’s SurveyMonkey platform. Data collection occurred between October and December 2022. Respondents were allowed to skip questions to mitigate forced recollection of uncomfortable or distressing experiences. The median completion time for the survey was 10.5 minutes.

### Study Participants

**Figure 1.**
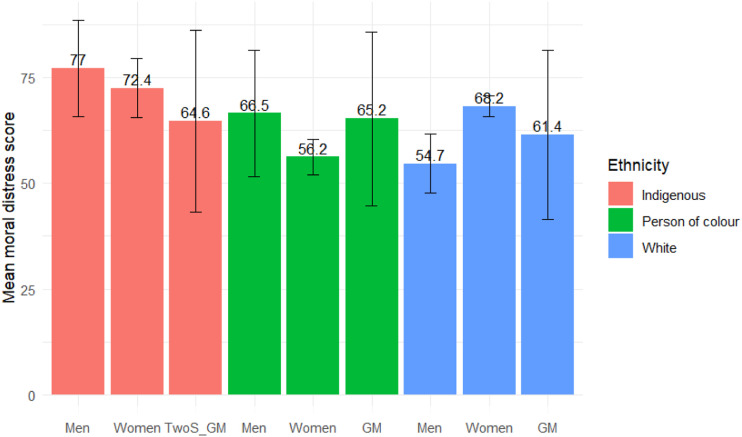
Mean moral distress score and 95% confidence intervals of groups classified by intersecting genders and racialized experiences. TwoS: two-spirit; GM: gender minority.

Our original sample included 3787 study participants. After excluding those who did not consent to the survey (*n* = 26), potential bots (*n* = 673)—determined by a survey completion time below three minutes—and participants who did not work in BC at the time of the survey (*n* = 170), 2918 healthcare workers in BC were considered eligible. Among them, 2104 were LTC workers. Our analytic sample included only 1678 participants who provided full outcome data (Figure 1), representing approximately 3.4% of all registered LTC workers in the province (Xiao, 2021). Participants who did not complete all questions were more likely to be older, foreign-born, or have full-time contracts, and less likely to work in one particular geographical BC health authority (i.e., Interior Health).

### Measures

The online survey included demographic questions, which covered age, gender (cis men, cis women, or gender minority), racialized experiences (indigenous, self-identifying as person of color, or white), and migratory information (Canadian-born or foreign-born). Occupational type of contract (full-time vs. part-time or casual), and place of work, which was later coded by health authority using information provided by the Province of British Columbia (2023). BC is divided into 5 health authorities, with three urban (Vancouver Coastal, Fraser & Island) divisions and two rural (Northern & Interior) areas.

Additionally, the questionnaire featured a shorter version (7-item) of the Measure of Moral Distress for Healthcare Professionals (MMD-HP) (Epstein et al., 2019), which has been validated for use in LTC settings. The selected questions referred to system-level root causes and breakdowns in workers’ interactions with patients and families (Epstein et al., 2019). Two additional moral distress questions were added, based on qualitative research with care workers in the province (Smith et al., 2022), to evaluate time-management for care of patients and self-care. All moral distress questions had two components: frequency and intensity, ranging from 0 to 4. Both components were multiplied to obtain a composite score (0–16), as recommended by the authors (Epstein et al., 2019). All composite scores add up to a range from 0 to 144. We also asked about distress-mitigation strategies (11 items). These questions were drawn from analysis of previous qualitative research on moral distress among healthcare workers in BC (Smith et al., 2022). This section also featured two components: frequency and effectiveness, ranging from 0 to 5. Respondents were asked to provide an estimate of the hypothetical effect if they had never implemented the coping strategy. The composite scores for each coping strategy ranged from 0 to 25. The overall distress mitigation score ranged from 0 to 275.

Finally, as part of the MMD-HP questionnaire, participants were asked whether they were “considering leaving [their] position now due to moral distress.” The available options were “No, I have never considered leaving or left a position,” “Yes, I considered leaving but did not leave,” and “Yes, I left a position.” The survey was piloted with eight healthcare workers, including those who did not speak English as a first language and representing a variety of genders, races, and ethnicities.

### Statistical Analysis

We described our sample using frequencies and proportions along with the mean score and standard deviation (SD) for the moral distress total score, stratifying our sample by health authority. Two-way intersections between gender and racialized experiences were created to analyze the distribution of levels of moral distress. We estimated means and 95% confidence intervals (CI) for each intersectional subgroup. Box plots were created to visually explore the variance of independent components of both the moral distress scale and the coping strategies. The Kruskal–Wallis chi-squared test and the Dunn test were performed to identify significant differences for each moral distress component and coping strategies between intersecting groups, adjusting adjusts the *p*-value for multiple comparisons using the Bonferroni method.

To explore the healthcare workers’ attributes that predicted the intention of leave work (outcome), we employed a Random Forest (RF) model. Previous studies have employed decision trees to perform intersection analysis (Mena & Bolte, 2021) because they can handle complex interactions between individual and group characteristics, they do not require assumptions of linearity, and summarize the overall importance of each characteristic in predicting a given outcome. RFs is a machine learning method that constructs multiple decision trees to improve the accuracy and stability of the predictive model, overcoming imbalances in the sampling of subgroups while avoiding overfitting (Mahendran et al., 2022).

We used all demographic and occupational variables at the person-level (i.e., excluding health authority), as well as moral distress and resilience scores as predictors. Both “Considered leaving” and “Left” were collapsed into a single option for the analysis. Then, we randomly divided the dataset (seed: “2223”) into a training set (70%) and a test set (30%). A training model of 500 trees was fitted using the “randomForest” package (Liaw & Wiener, 2002), applying 10-fold cross-validation. Subsequently, the model was adjusted for an optimal number of variables evaluated at each split or “mtry” (range 1–10), maximum nodes (range 5–25), and number of trees (250, 300, 350, 400, 450, 500, 550, 600, 800, 1000, and 2000). The optimized model with a mtry of, a maximum node of 15, and 800 trees was evaluated against the test set using the “caret” package (Kuhn, 2008). Only complete cases were included in the RF analysis. All analyses were performed using R v 4.3.0.

### Positionality

Intersectional research requires reflexivity on the part of the researchers (Bowleg, 2013). Here, we recognize that while we are a diverse team in terms of race/ethnicity, and gender—as well as lived experiences—we occupy positions of relative privilege and power, most notably in terms of our academic expertise and position as those who collect and analyze data about those experiencing hardship and discrimination. In particular, we are at risk of the privilege hazard, “the phenomenon that makes those who occupy the most privileged positions among us – those with good educations, respected credentials, and professional accolades – so poorly equipped to recognize instances of oppression in the world” (D’Ignazio & Klein, 2020). In other words, our academic expertise, while privileged in the mainstream knowledge economy, is actually a disadvantage, compared to the lived experience of research participants. We try to mitigate this hazard by first acknowledging it, and then grounding any recommendations in the experiences participants generously shared with us.

It is further important to note that our team does not include anyone who identifies as Indigenous or an Indigenous health organization. This is a notable gap considering some of the results that follow and the context of colonization in Canada. Recognizing this, we have shared results with all Indigenous participants who consented to follow-up inviting feedback on results, dissemination, and next steps. We further contextualize findings, in the discussion, within the context of colonization and discrimination, noting this study points to the need for further, Indigenous led, research on the LTC workforce.

## Results

### Sample Characteristics

Table 1 shows the sample characteristics stratified by health authority. The majority (79.5%) of the sample identified as women, however, there was significant variation by health authority, ranging from 82% in Vancouver Coastal Health (VCH) to 94.6% in Northern Health. There was also a significant variation in age, with higher participation from groups under 30 in the Island (32.9%) and Northern (41.9%) health authorities. Furthermore, the two rural health areas had a significantly higher proportion of people who identified as Indigenous or White. A much-higher proportion of foreign-born workers worked in the mostly urban VCH and Fraser, also fewer people had considered leaving in these regions which, along with the Island Health Authority, had lower moral distress mean scores. Higher distress mitigation scores were found in participants from the Northern area. Workers from Interior Health had significantly higher moral distress levels than Vancouver Coastal (*p* < .01), Vancouver Island (*p* = .02), and Fraser (*p* < .01) whereas LTC workers from the Northern Health Authority had higher moral distress levels than workers from Vancouver Coastal (*p* = .02). Northern workers also had significantly higher levels of distress mitigation compared to all other health authority participants (*p* < .05 in all cases).

**Table 1.** Characteristics of Long-Term Care Workers in British Columbia by Health Authority^a^ Who Participated in the Survey.

	Northern (*n* = 74)	Interior (*n* = 403)	Vancouver Coastal (*n* = 222)	Vancouver Island (*n* = 341)	Fraser (*n* = 305)	Overall^a^ (*n* = 1678)
Age (%)						
Under 30	31 (41.9)	71 (17.8)	32 (14.4)	112 (32.9)	51 (16.8)	398 (23.8)
30–39	18 (24.3)	96 (24.0)	69 (31.1)	82 (24.1)	100 (32.9)	473 (28.3)
40–49	14 (18.9)	102 (25.5)	72 (32.4)	70 (20.6)	72 (23.7)	389 (23.3)
50–59	8 (10.8)	99 (24.8)	40 (18.0)	57 (16.8)	63 (20.7)	306 (18.3)
Over 60	3 (4.1)	32 (8.0)	9 (4.1)	19 (5.6)	18 (5.9)	106 (6.3)
Gender (%)						
Cis man	2 (2.7)	32 (8.0)	34 (15.4)	37 (10.9)	28 (9.3)	163 (10.6)
Cis woman	70 (94.6)	363 (90.8)	182 (82.4)	298 (87.6)	271 (89.7)	1334 (86.7)
Gender minority^b^	2 (2.7)	5 (1.2)	5 (2.3)	5 (1.5)	3 (1.0)	41 (2.7)
Racialized experiences (%)						
Indigenous^c^	10 (13.5)	49 (12.2)	9 (4.1)	33 (9.7)	21 (6.9)	158 (9.4)
People of color	11 (14.9)	39 (9.7)	97 (43.9)	86 (25.3)	103 (33.9)	534 (31.9)
White	53 (71.6)	314 (78.1)	115 (52.0)	221 (65.0)	180 (59.2)	981 (58.6)
Foreign-born (%)	17 (23.3)	81 (20.1)	131 (59.0)	65 (19.1)	168 (55.1)	660 (39.4)
Full-time employee (%)	40 (54.1)	203 (50.4)	131 (59.0)	201 (58.9)	169 (55.4)	908 (54.1)
Moral distress score [mean (SD)]	73.95 (31.25)	73.16 (37.37)	58.07 (37.72)	64.55 (38.29)	62.08 (40.29)	61.55 (38.49)
Distress mitigation score [mean (SD)]	96.96 (49.67)	72.99 (37.60)	77.45 (46.02)	74.79 (38.42)	80.15 (44.67)	76.39 (45.02)
Considering leaving (%)						
No	16 (21.6)	120 (29.8)	95 (42.8)	86 (25.2)	126 (41.3)	597 (35.6)
Yes, but has not	53 (71.6)	240 (59.6)	116 (52.3)	227 (66.6)	165 (54.1)	965 (57.5)
Left	5 (6.8)	43 (10.7)	11 (5.0)	28 (8.2)	14 (4.6)	116 (6.9)

^a^Missing data for all variables was below .5%, except for health authority (19.85%), gender (8.34%), and resilience score (4.41%).

^b^Includes trans and non-binary identities, and two-spirit indigenous people.

^c^People who selected both indigenous and non-white were classified as indigenous.

### Moral Distress

Indigenous men had the highest mean distress scores (77.0, 95% CI: 65.7–88.4), this score was significantly higher than that of White men (54.7, 95% CI: 47.7–61.6), and women of color (56.2, 95% CI: 52.0–60.3). They, in turn, had significantly lower scores than White (68.2, 95% CI: 65.7–70.7) and Indigenous women (72.4, 95% CI: 65.4–79.4) (Figure 1). Overall, it was hard to evaluate the distress score for gender minority groups as their sample size was extremely small (Table 2). Even the aggregated score for all gender minority participants (64.0, 95% CI: 52.9–75.1) was not significantly different from other groups.

**Table 2.** Moral Distress Score by Groups of Intersecting Gender and Potential Past Racialized Experiences.

Intersecting Identity	*n*	Mean Moral Distress Score	95% Confidence Interval (CI)
Indigenous men	36	77.03	65.65–88.40
Indigenous women	106	72.43	65.44–79.43
Indigenous gender minorities	14	64.64	43.10–86.18
Men of color	32	66.47	51.47–81.46
Women of color	353	56.16	52.05–60.27
Gender minorities of color	16	65.25	44.77–85.73
White men	95	54.65	47.69–61.62
White women	870	68.17	65.67–70.67
White gender minorities	11	61.36	41.38–81.35

Box plots comparing the different distress components by gender and racialized experiences are available as supplementary data (Figure S1). Overall, requirements for caring for more patients than one can safely care for was the most distressing professional experience across groups, with significantly increased levels of distress for White and Indigenous women compared to White men and women of color. The same pattern was found when examining lack of time for self-care.

Indigenous men reported significantly higher levels of stress due to feeling unqualified than White men and women and women of color. In turn, Indigenous and White women reported higher levels of stress because their “patient care suffered because of a lack of provider continuity” compared to White men. Requirements to “work with other care providers who are not as competent as patient care requires,” and lack of administrative support, were more distressing for White and Indigenous women than for women of color. Experiences of low-quality patient care due to poor team communication and compromised patient care due to lack of resources or equipment were more distressing for White women compared to women of color. Finally, lack of time to provide for patient needs was more distressing for Indigenous men and women as well as for White women compared to women of color (supplementary data).

### Distress Mitigation

**Figure 2.**
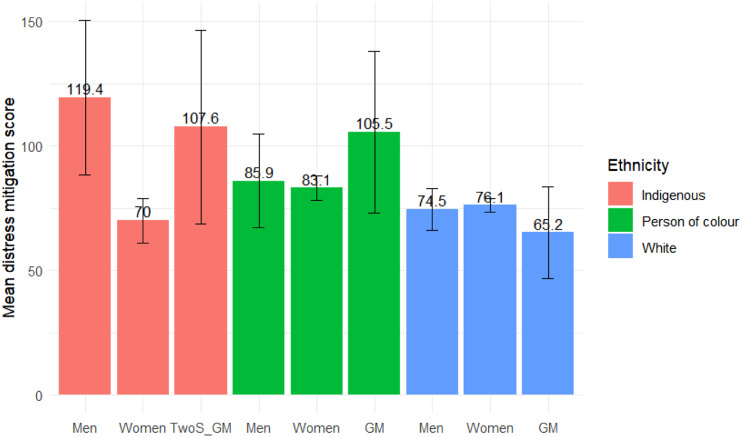
Mean distress mitigation score and 95% confidence intervals of groups classified by intersecting genders and potential past racialized experiences. TwoS: two-spirit; GM: gender minority.

Indigenous men also had the highest mean distress mitigation scores for all groups (119.0, 95% CI: 88.5% to 150.0), this score was significantly higher than that of all White groups, including men (74.5, 95% CI: 66.1–82.9), women (76.1, 95% CI: 73.3–79.0), and gender minorities (65.2, 95% CI: 46.6–83.8). It was also significantly higher than that of Indigenous women (70.0, 95% CI: 61.1–78.9) (Figure 2 and Table 3). The component analysis reflects this overall trend for most coping strategies (Figure S2).

**Table 3.** Distress Mitigation Score by Groups of Intersecting Gender and Potential Past Racialized Experiences.

Intersecting Identity	*n*	Mean Resilience Score	95% Confidence Interval (CI)
Indigenous men	33	119.3636	88.46–150.27
Indigenous women	102	69.9902	61.09–78.89
Indigenous gender minorities	13	107.6154	68.71–146.52
Men of color	29	85.93103	67.22–104.64
Women of color	325	83.07692	78.02–88.14
Gender minorities of color	14	105.5	72.98–138.02
White men	92	74.53261	66.13–82.93
White women	840	76.12619	73.27–78.98
White gender minorities	11	65.18182	46.60–83.76

Spending time with family and spending time outdoors were deemed the most useful distress mitigation strategies among all groups, whereas counseling and the supervisor phone line were deemed the least helpful. White women found more relief in spending time outdoors compared to women of color. In turn, women of color found spending time with their family more relieving than Indigenous women and White men. Indigenous men, on the other hand, reported more relief from peer support and mindfulness exercises compared to Indigenous women and White men and women; women of color also found mindfulness exercises more relieving than White women. Religion has more relieving effects on Indigenous men, Indigenous gender minorities and women of color, compared to White men and women and Indigenous women. Among gender minorities, religion was more effective for Indigenous than White participants and for gender minorities of color compared to White women (supplementary data).

Supervisor support was more useful for Indigenous men and women of color compared to White and Indigenous women. Gender minorities of color also found more relief in seeking supervisor support than Indigenous women. Indigenous men and gender minorities found more relief in using the support phone line compared to Indigenous women, men and women of color, and White men and women. Additionally, women of color and White men also found more relief in the phone line compared to White women. Wider differences were found with respect to counseling/therapy, where Indigenous men found it significantly more relieving than Indigenous women and men of color, but Indigenous women (as well as Indigenous gender minorities) also found it significantly more relieving than White women and women of color. Gender minorities of color also found more relief in counselling/therapy compared to White men and women and men and women of color. White and Indigenous men and Indigenous gender minorities coped better through self-medication compared to White women and women of color. Among people of color, gender minorities found more relief in self-medication than women. Finally, Indigenous gender minorities had higher levels of self-medication relief than men of color (supplementary data).

### Intention to Leave Employment

**Figure 3.**
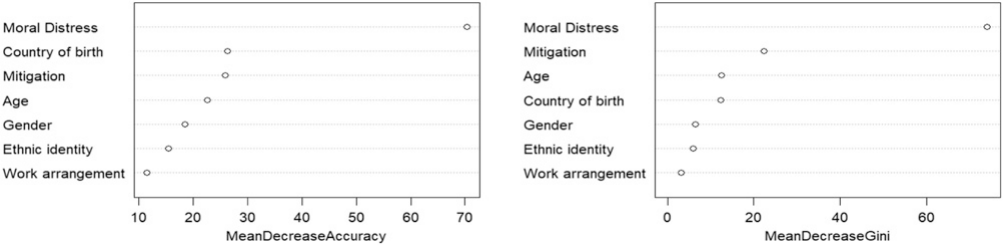
Variable importance for predicting intention to leave employment measures based on mean decrease in accuracy (left) and Gini index (right).

Overall, 57% of participants were considering leaving their position and 6.7% had left their position due to moral distress. 73.5% of Indigenous women were considering leaving their position, compared to 60.7% of women of color and 54.5% of White women. Among men, the proportion was higher for people of color (69.0%) than for Indigenous (46.9%) and White men (48.9%). Gender minority groups had similar proportions for Indigenous (58.3%), people of color (57.1%), and White participants (54.5%). The random forest model had an accuracy of 73.54% (95% CI: 69.19%–77.58%) predicting this outcome. Its sensitivity was 45.27% and its specificity 87.58%. The most important predictor of intention to leave employment—meaning the variable which was more likely to split groups to help them organize between those who intended to leave work and those who did not—was the moral distress score (54.81%), followed by the distress mitigation score (15.92%), age (8.97%), and country of birth (Canada vs. foreign-born) (8.82%). All other variables accounted for less than 5% of the prediction each (Figure 3). As an example, Supplementary Figure 3 shows a sample tree where the distress mitigation score (cut off: 183) was identified as the root of the three and later used to split groups in other branches. Similarly, stress scores (“mdi”) split the sample multiple times at the furthest branch. When analyzing the variable importance by mean decrease of accuracy, country of birth showed a higher overall impact, overtaking age, and the distress mitigation score, but not within classes or their intersections.

## Discussion

This paper contributes an intersectional analysis to the growing literature on moral distress among LTC providers. The differences across racial and gender identity groups, in both sources of distress and preferred mitigation strategies, suggest the need for tailored interventions to address moral distress among LTC providers. Among racial identities, Indigenous people were the only group where significant differences in mitigation scores were found. While Indigenous men were, on average, the most distressed group, they also had higher mean distress mitigation scores. In other words, they identified effective coping strategies, which likely influenced the low proportion on intention to leave work across all groups (46.9%). In turn, Indigenous women were also highly burdened by moral distress, but had a lower mean score for distress mitigation strategies, and the proportion of Indigenous women considering leaving their position was the highest among all groups (73.5%). Qualitative research including Indigenous LTC care providers suggests heightened moral distress might reflect Indigenous cultural values around respect and care for the elderly (Booi et al., 2021). Settler-colonial culture in Canada often expresses the opposite, being characterized by agism and a lack of valuing of care work (Pijl-Zieber et al., 2008), and it is possible that working in a colonial cultural context contributes to moral distress for Indigenous LTC providers. Increased risk of racism and discrimination at work may also be a factor (GOvBC, 2020). Further, preferably Indigenous-led, research is needed to better understand these results and inform community led responses.

While not analyzed directly, findings also suggest a tangible impact of the mitigation strategies, a finding which is reinforced by our random forest model, where the mitigation score ranked second among all predictors of the intention to leave the workplace. They also raise questions about gendered differences in access to mitigation strategies—for example, do lower distress mitigation scores among women indicate structural barriers to coping with distress?

The most distressing experiences to those who make up the bulk of the workforce—white women and women of color—were those related to workload, lack of time with residents, and lack of time for self. Time with family was the preferred distress mitigation strategy for both groups. While previous studies have rarely analyzed for race/ethnicity and gender, they have noted similar overall findings in terms of LTC providers experiencing work overload and distress related to resident care (Booi et al., 2021; Braedley et al., 2018; Pijl-Zieber et al., 2008), with research with women care providers, particularly noting the tension between work burdens, time with family, and for self-care (Smith et al., 2022).

Healthcare workers in more rural health authorities, namely Northern Health and Interior Health, report higher moral distress scores than those in more urban and central locations, such as Vancouver Coastal, Fraser Health, and Island Health. Notably, the rural health authorities are experiencing the greatest staffing shortages (Canadian Institute for Health Information, 2023) which may partly explain this disparity and demonstrates an urgent need to address the causes of moral distress among LTC providers in rural communities.

While there is variation in moral distress scores along racial and gender axis, there is also notable overlap. Requirements for caring for more patients than one can safely care for was the most distressing professional experience across groups, with lack of time for self-care also being a shared cause of moral distress. This suggests that time poverty—not having the time to do what needs to be done—may be a prominent determinant of moral distress, with staffing shortages preventing LTC providers from providing the care residents and they themselves need. Previous research has similarly linked time poverty to lower well-being, physical health and productivity (Giurge et al., 2020). Notably, preferred mitigation strategies, such as time outdoors and with family, also require time, as opposed to less popular interventions like phone lines and counselling.

The relationship between moral distress score and intention to leave employment confirms previous research that indicates a relationship between moral distress and attrition (Epstein et al., 2019). The intention to leave employment (57.5%) is notably higher here than many other moral distress studies—for example one review found intention to leave employment due to moral distress ranged from 10% to 38% (Karakachian & Colbert, 2019)—but similar to results from a survey prepared by a union representing those who work in LTC in the province, which found over 75% healthcare workers were experiencing burnout and one in three was considering leaving the profession (HEU, 2022). This raises concerns for the LTC sector in BC, which continues to struggle with staffing shortages and continued straining impacts of the COVID-19 pandemic.

Furthermore, higher rates of intention to leave employment among women reflect racial inequities, with Indigenous and people of color providers more likely to leave than those who identify as White. Among men, those identifying as a person of color had higher rates of intention to leave than White and Indigenous men. The relationship between country of birth and intention to leave employment merits further exploration as those participants who were foreign-born were, in general, less likely to intend to leave their position. While we inquired about country of birth, we did not include questions about legal status and it is possible that, for some participants, the conditions of their employment contracts and work permits within Canada could potentially diminish their likelihood to consider resignation, adding an additional layer of complexity to the study’s findings.

While limited by a particular focus on gender and race/ethnicity, this represents one of the earliest efforts to apply intersectional analysis in the exploration of moral distress. Our study also piloted a distress mitigation scale in this survey, adding an additional tool to the moral distress toolbox. The scale both acknowledges the limits of much moral distress research (related to lack of focus on agency and supportive interventions) and embodies the motivation underpinning intersectional research approaches. Beyond analyzing how various social positions reflect inequities, our work also informs strategies to dismantle them.

Our study was grounded in a conceptual framework and theoretical underpinnings rooted in intersectional feminism, which guided our research questions and expectations. We did not include a pre-established set of a priori directional hypotheses, while we recognize the value of articulating specific hypotheses to enhance research transparency and analytical rigor, this study served as an exploratory endeavor. In future research, the formulation and testing of explicit hypotheses could contribute to further refining and validating our findings.

The high specificity of our results suggests there is a high degree of confidence in the accuracy of this assessment. This can be valuable for ensuring that interventions are appropriately targeted toward those who genuinely require support. However, the low sensitivity implies that our tool may fail to identify a significant portion of LTC workers who are silently experiencing moral distress. In practical terms, our findings highlight the need for a more comprehensive and nuanced approach to addressing moral distress in LTC settings. Rather than relying solely on a single screening tool with limited sensitivity, it may be beneficial to implement a multi-faceted approach. This could include regular surveys or assessments to capture a broader spectrum of distress experiences.

Our study also has some limitations, we have not explored in detail how racism, sexism, colonialism, and other forms of discrimination may interact with experiences of moral distress—an area that requires further research. Similarly, due to sample size, our study was prone to type II error, particularly regarding LTC providers identifying as gender minorities, suggesting the experiences of this population group may be better captured through a targeted survey and/or qualitative methods, such as focus groups or interviews. Given our sample was not randomly selected, it is possible that some findings may not be applicable across the entire LTC workforce in BC. The complete case analysis also might introduce similar biases. Furthermore, we analyzed the intention to leave work the job using a single-item question, which may not capture the complexity and depth of this construct. Hence this finding should be interpreted with caution, as it may be less stable than findings based on more systematically measured constructs. Finally, the random forest model had low sensitivity, meaning that the model was better at identifying those not at risk than those who intended to leave their position. Considering these constraints, our findings should be interpreted within the context of future research conducted on similar groups.

## Conclusion

The consequences of unaddressed moral distress extend beyond individual healthcare providers to impact the entire health system. A workforce plagued by moral distress is more likely to experience high turnover rates and difficulties in recruitment. This, in turn, can contribute to decreased efficiency, productivity, and ultimately, suboptimal patient outcomes. Our findings related to moral distress point towards the need for structural changes, such as increased staffing and adaptive schedules, to address the complex challenges faced by LTC providers. Furthermore, differences across groups point to the need to prioritize interventions for those most affected, which this study identifies as Indigenous care aids, as well as women care aids.

The findings related to distress mitigation strategies support structural approaches, particularly considering that the preferred and most efficacious mitigation strategies across all groups primarily require time, which requires reducing work hours. While recognizing that building a sustainable LTC workforce is a long-term challenge, more immediate interventions might include identifying how shifts might better correspond to family schedules to allow time with family. Workplace flexibility has been found to mitigate the effects of burnout among healthcare workers (Maglalang et al., 2021). Other interventions might include paid time for self-care activities, such as exercise, and creating outdoor spaces LTC providers can access at or near work. Again, priority might be given to the preferences of those with lower distress mitigation scores, such as Indigenous women. Further research and collaboration with stakeholders are warranted to develop comprehensive strategies to address differential impacts on LTC providers.

## Supplemental Material

Supplemental Material - An Intersectional Analysis of Moral Distress and Intention to Leave Employment Among Long-Term Care Providers in British Columbia Supplemental Material for An Intersectional Analysis of Moral Distress and Intention to Leave Employment Among Long-Term Care Providers in British Columbia by Julia Smith, Muhammad Haaris Tiwana, Hasina Samji, Rosemary Morgan, Simran Purewal, and Jorge Andres Delgado-Ron in Journal of Aging and Health.

## References

[bibr1-08982643231212981] Bauer G. R. Churchill S. M. Mahendran M. Walwyn C. Lizotte D. Villa-Rueda A. A. (2021). Intersectionality in quantitative research: A systematic review of its emergence and applications of theory and methods. SSM - Population Health, 14, 100798. 10.1016/j.ssmph.2021.100798 33997247 PMC8095182

[bibr2-08982643231212981] Booi L. Sixsmith J. Chaudhury H. O’Connor D. Young M. Sixsmith A. (2021). ‘I wouldn’t choose this work again’: Perspectives and experiences of care aides in long-term residential care. Journal of Advanced Nursing, 77(9), 3842–3852. 10.1111/jan.14948 34235778

[bibr3-08982643231212981] Bourgeault I. L. Atanackovic J. Rashid A. Parpia R. (2010). Relations between immigrant care workers and older persons in home and long-term care. Canadian Journal on Aging/La Revue Canadienne Du Vieillissement, 29(1), 109–118. 10.1017/S0714980809990407 20202269

[bibr4-08982643231212981] Bowleg L. (2013). “Once you’ve blended the cake, you can’t take the parts back to the main ingredients”: Black gay and bisexual men’s descriptions and experiences of intersectionality. Sex Roles, 68(11–12), 754–767. 10.1007/s11199-012-0152-4

[bibr5-08982643231212981] Braedley S. Owusu P. Przednowek A. Armstrong P. (2018). We’re told, ‘suck it up’: Long-term care workers’ psychological health and safety. Ageing International, 43(1), 91–109. 10.1007/s12126-017-9288-4

[bibr6-08982643231212981] Canadian Association for Long Term Care . (2023). Addressing the staffing emergency in long-term care in Canada. CALTC. https://www.ourcommons.ca/Content/Committee/441/HUMA/Brief/BR11698352/br-external/CanadianAssociationForLongTermCare-e.pdf

[bibr42-08982643231212981] Canadian Institute for Health Information . (2023). *Healthworkforce: Healthcare providers.* CIHI. https://www.cihi.ca/en/topics/health-workforce/health-care-providers?keyword=aid&items_per_page=50

[bibr7-08982643231212981] Collins P. H. (2015). Intersectionality’s definitional dilemmas. Annual Review of Sociology, 41(1), 1–20. 10.1146/annurev-soc-073014-112142

[bibr8-08982643231212981] Crenshaw K. (1997). Demarginalizing the Intersection of Race and Sex: A Black Feminist Critique of Antidiscrimination Doctrine, Feminist Theory and Antiracist Politics. *University of Chicago Legal Forum**, *1989(1), 139–167. https://chicagounbound.uchicago.edu/cgi/viewcontent.cgi?article=1052&context=uclf

[bibr39-08982643231212981] D’Ignazio C. Klein L. F. (2020) (In press). The Power Chapter. In Data Feminism. MIT Press.

[bibr9-08982643231212981] Duan Y. Iaconi A. Song Y. Norton P. G. Squires J. E. Keefe J. Cummings G. G. Estabrooks C. A. (2020). Care aides working multiple jobs: Considerations for staffing policies in long-term care homes during and after the COVID-19 pandemic. Journal of the American Medical Directors Association, 21(10), 1390–1391. 10.1016/J.JAMDA.2020.07.036 32893137 PMC7472068

[bibr10-08982643231212981] Epstein E. G. Whitehead P. B. Prompahakul C. Thacker L. R. Hamric A. B. (2019). Enhancing understanding of moral distress: The measure of moral distress for health care professionals. AJOB Empirical Bioethics, 10(2), 113–124. 10.1080/23294515.2019.1586008 31002584

[bibr11-08982643231212981] Estabrooks C. A. Straus S. E. Flood C. M. Keefe J. Armstrong P. Donner G. J. Boscart V. Ducharme F. Silvius J. L. Wolfson M. C. (2020). Restoring trust: COVID-19 and the future of long-term care in Canada. Facets, 5(1), 651–691. 10.1139/facets-2020-0056

[bibr12-08982643231212981] Giurge L. M. Whillans A. V. West C. (2020). Why time poverty matters for individuals, organisations and nations. Nature Human Behaviour, 4(10), 993–1003. Article 10. 10.1038/s41562-020-0920-z 32747805

[bibr13-08982643231212981] GOvBC . (2020). Plain sight: Addressing indigenous-specific racism and discrimination in B.C. health care. Government of BC. https://engage.gov.bc.ca/app/uploads/sites/613/2020/11/In-Plain-Sight-Summary-Report.pdf

[bibr14-08982643231212981] Greason M. (2020). Ethical reasoning and moral distress in social care among long-term care staff. Journal of Bioethical Inquiry, 17(2), 283–295. 10.1007/s11673-020-09974-x 32297016

[bibr40-08982643231212981] Hart S. E. (2005) (In this issue). Hospital Ethical Climates and Registered Nurses’ Turnover Intentions. Journal of Nursing Scholarship, 37(2), 173–177. 10.1111/j.1547-5069.2005.00030.x 15960062

[bibr15-08982643231212981] Havaei F. Tang X. Smith P. Boamah S. A. Frankfurter C. (2022). The association between mental health symptoms and quality and safety of patient care before and during COVID-19 among Canadian nurses. Healthcare, 10(2), 314. 10.3390/HEALTHCARE10020314 35206927 PMC8871834

[bibr16-08982643231212981] HEU . (2022). Poll: Two years into pandemic, one in three health care workers likely to quit. HEU. https://www.heu.org/news/media-release/poll-two-years-pandemic-one-three-health-care-workers-likely-quit

[bibr17-08982643231212981] Jameton A. (2017). What moral distress in nursing history could suggest about the future of health care. AMA Journal of Ethics, 19(6), 617–628. 10.1001/journalofethics.2017.19.6.mhst1-1706 28644792

[bibr18-08982643231212981] Karakachian A. Colbert A. (2019). Nurses’ moral distress, burnout, and intentions to leave: An integrative review. Journal of Forensic Nursing, 15(3), 133–142. 10.1097/JFN.0000000000000249 31436681

[bibr19-08982643231212981] Kuhn M. (2008). Building predictive models in R using the caret package. Journal of Statistical Software, 28(5), 1–26. 10.18637/jss.v028.i0527774042

[bibr20-08982643231212981] Lakusta W. (2018). Employer perspectives on personal support worker recruitment and retention. Health Force Ontario.

[bibr21-08982643231212981] Liaw A. Wiener M. (2002). Classification and regression by randomForest. R News, 2(3), 18–22.

[bibr22-08982643231212981] Maglalang D. D. Sorensen G. Hopcia K. Hashimoto D. M. Katigbak C. Pandey S. Takeuchi D. Sabbath E. L. (2021). Job and family demands and burnout among healthcare workers: The moderating role of workplace flexibility. SSM - Population Health, 14, 100802. 10.1016/j.ssmph.2021.100802 33997249 PMC8102798

[bibr23-08982643231212981] Mahendran M. Lizotte D. Bauer G. R. (2022). Describing intersectional health outcomes: An evaluation of data analysis methods. Epidemiology, 33(3), 395–405. 10.1097/EDE.0000000000001466 35213512 PMC8983950

[bibr24-08982643231212981] McCarthy J. Gastmans C. (2015). Moral distress: A review of the argument-based nursing ethics literature. Nursing Ethics, 22(1), 131–152. 10.1177/0969733014557139 25505098

[bibr25-08982643231212981] Mena E. Bolte G. Advance Gender Study Group . (2021). CART-analysis embedded in social theory: A case study comparing quantitative data analysis strategies for intersectionality-based public health monitoring within and beyond the binaries. SSM - Population Health, 13, 100722. 10.1016/j.ssmph.2020.100722 33385059 PMC7772559

[bibr26-08982643231212981] Office of the Seniors Advocate . (2020). A billion reasons to care. Office of the Seniors Advocate.

[bibr27-08982643231212981] Pijl-Zieber E. Hagen B. Armstrong-Esther C. Hall B. Akins L. Stingl M. (2008). Moral distress: An emerging problem for nurses in long-term care? Quality in Ageing and Older Adults, 9(2), 39–48. 10.1108/14717794200800013

[bibr28-08982643231212981] Province of British Columbia . (2023). Regional health authorities. Province of British Columbia. https://www2.gov.bc.ca/gov/content/health/about-bc-s-health-care-system/partners/health-authorities/regional-health-authorities

[bibr29-08982643231212981] Rushton C. H. Caldwell M. Kurtz M. (2016). CE: Moral distress: A catalyst in building moral resilience. American Journal of Nursing, 116(7), 40–49. 10.1097/01.NAJ.0000484933.40476.5b 27294668

[bibr30-08982643231212981] Smith J. Korzuchowski A. Memmott C. Oveisi N. Tan H.-L. Morgan R. (2023). Double distress: Women healthcare providers and moral distress during COVID-19. Nursing Ethics, 30(1), 46–57. 10.1177/09697330221114329 36260872 PMC9582741

[bibr31-08982643231212981] Statistics Canada, SC . (2020). The contribution of immigrants and population groups designated as visible minorities to nurse aide, orderly and patient service associate occupations. Statistics Canada. https://www150.statcan.gc.ca/n1/pub/45-28-0001/2020001/article/00036-eng.htm

[bibr32-08982643231212981] Statistics Canada, SC (2022, March 6). Experiences of health care workers during the COVID-19 pandemic, September to November 2021. Statistics Canada. https://www150.statcan.gc.ca/n1/daily-quotidien/220603/dq220603a-eng.htm

[bibr33-08982643231212981] Syed I. U. (2020). Racism, racialization, and health equity in Canadian residential long term care: A case study in Toronto. Social Science & Medicine (1982), 265, 113524. 10.1016/j.socscimed.2020.113524 33228980

[bibr35-08982643231212981] Xiao X. (2021, October 13). Ninety-five percent of workers in BC long-term care homes have been vaccinated as mandate kicks in.* The globe and mail**. * *https://www.theglobeandmail.com/canada/british-columbia/article-ninety-five-percent-of-workers-in-bc-long-term-care-homes-have-been/*

