# Supplementary material for: An Intersectional Analysis of Moral Distress and Intention to Leave Employment Among Long-Term Care Providers in British Columbia
Source: J Aging Health. 2023 Nov 9;36(10):689–99. doi: 10.1177/08982643231212981 (PMC11531082; doi:10.1177/08982643231212981)
Supplement: Supplemental Material - An Intersectional Analysis of Moral Distress and Intention to Leave Employment Among Long-Term Care Providers in British Columbia [file sj-pdf-1-jah-10.1177_08982643231212981.pdf]

Supplementary figures

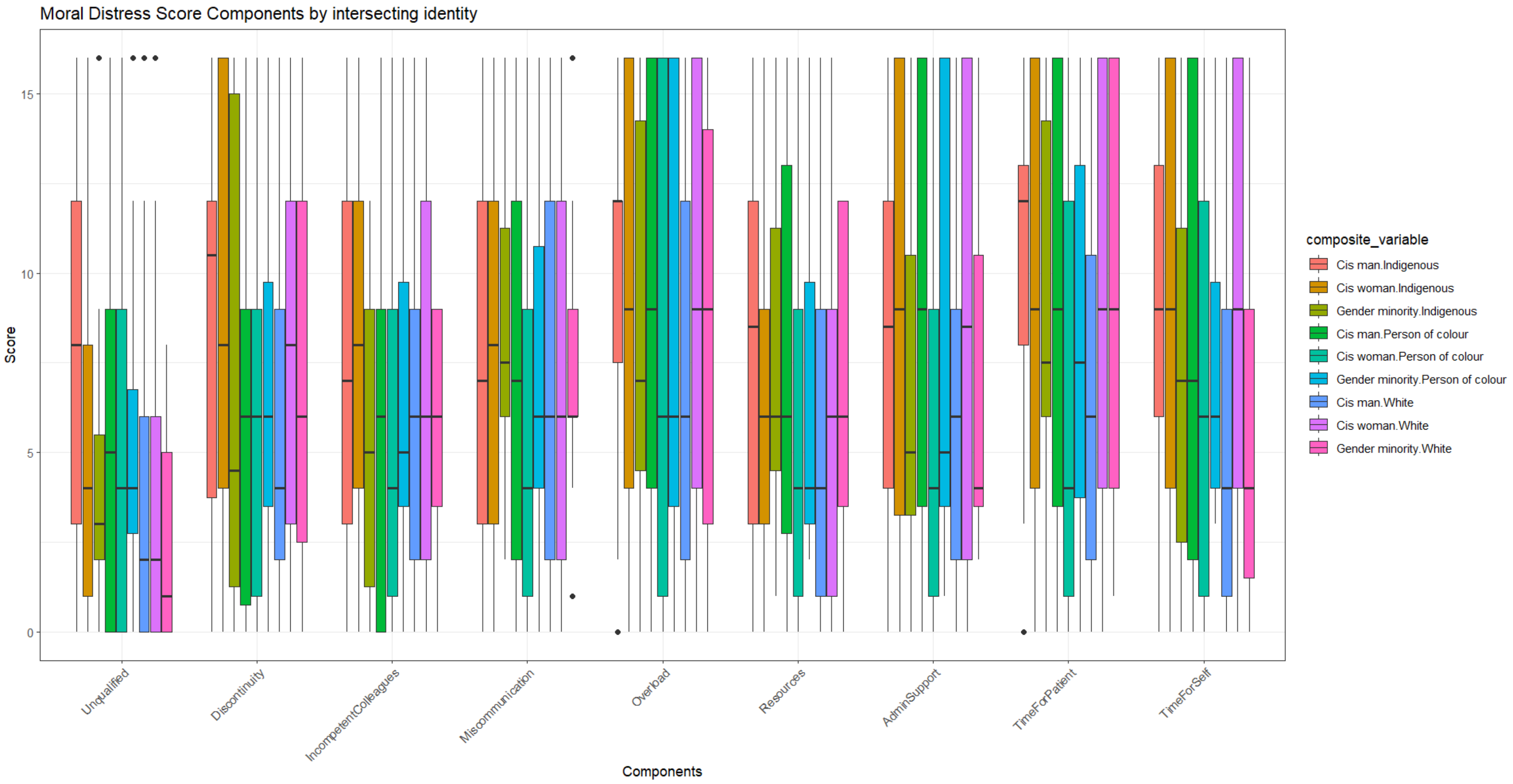

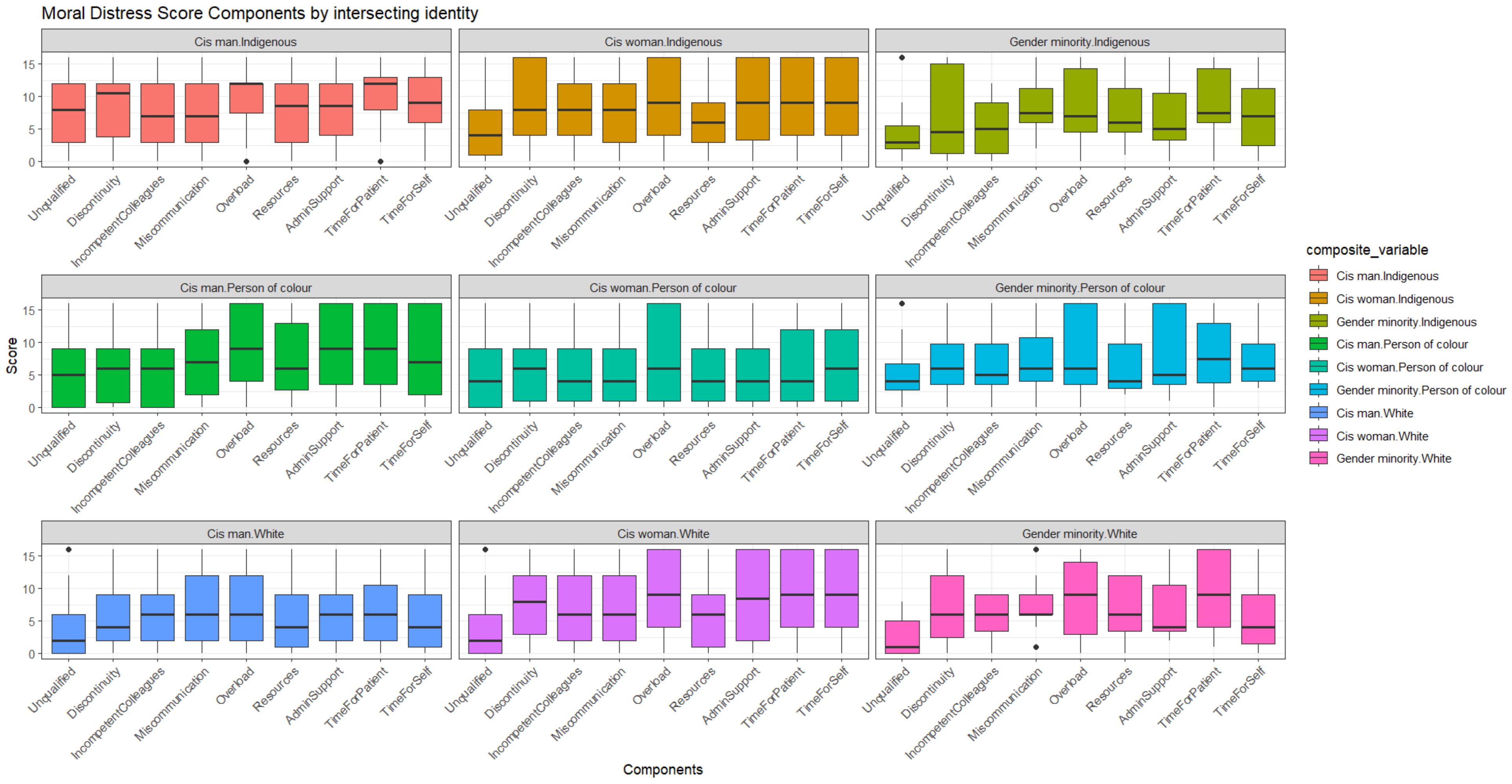
Moral Distress Score Components by intersecting identity
Cis man.Indigenous
Cis woman.Indigenous
Gender minority.Indigenous
Cis man.Person of colour
Cis woman.Person of colour
Gender minority.Person of colour
Cis man.White
Cis woman.White
Gender minority.White
Score
0
5
10
15
Unqualified
Discontinuity
IncompetentColleagues
Miscommunication
Overload
Resources
AdminSupport
TimeForPatient
TimeForSelf
Components
composite_variable
Cis man.Indigenous
Cis woman.Indigenous
Gender minority.Indigenous
Cis man.Person of colour
Cis woman.Person of colour
Gender minority.Person of colour
Cis man.White
Cis woman.White
Gender minority.White

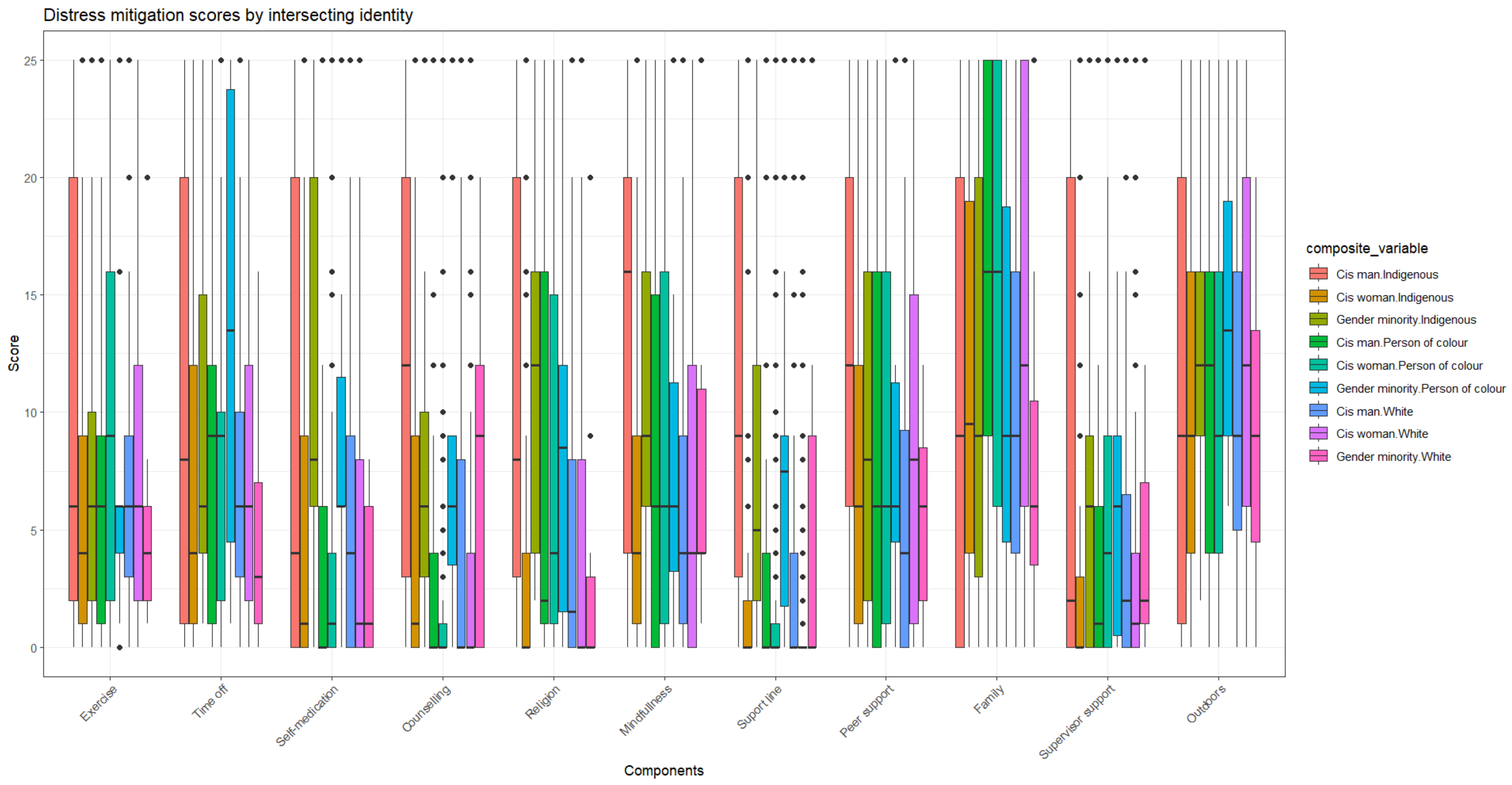

Distress mitigation scores by intersecting identity
Score
25
20
15
10
5
0
Exercise
Time off
Self-medication
Counselling
Religion
Mindfulness
Suport line
Peer support
Family
Supervisor support
Outdoors
Components
composite_variable
Cis man.Indigenous
Cis woman.Indigenous
Gender minority.Indigenous
Cis man.Person of colour
Cis woman.Person of colour
Gender minority.Person of colour
Cis man.White
Cis woman.White
Gender minority.White

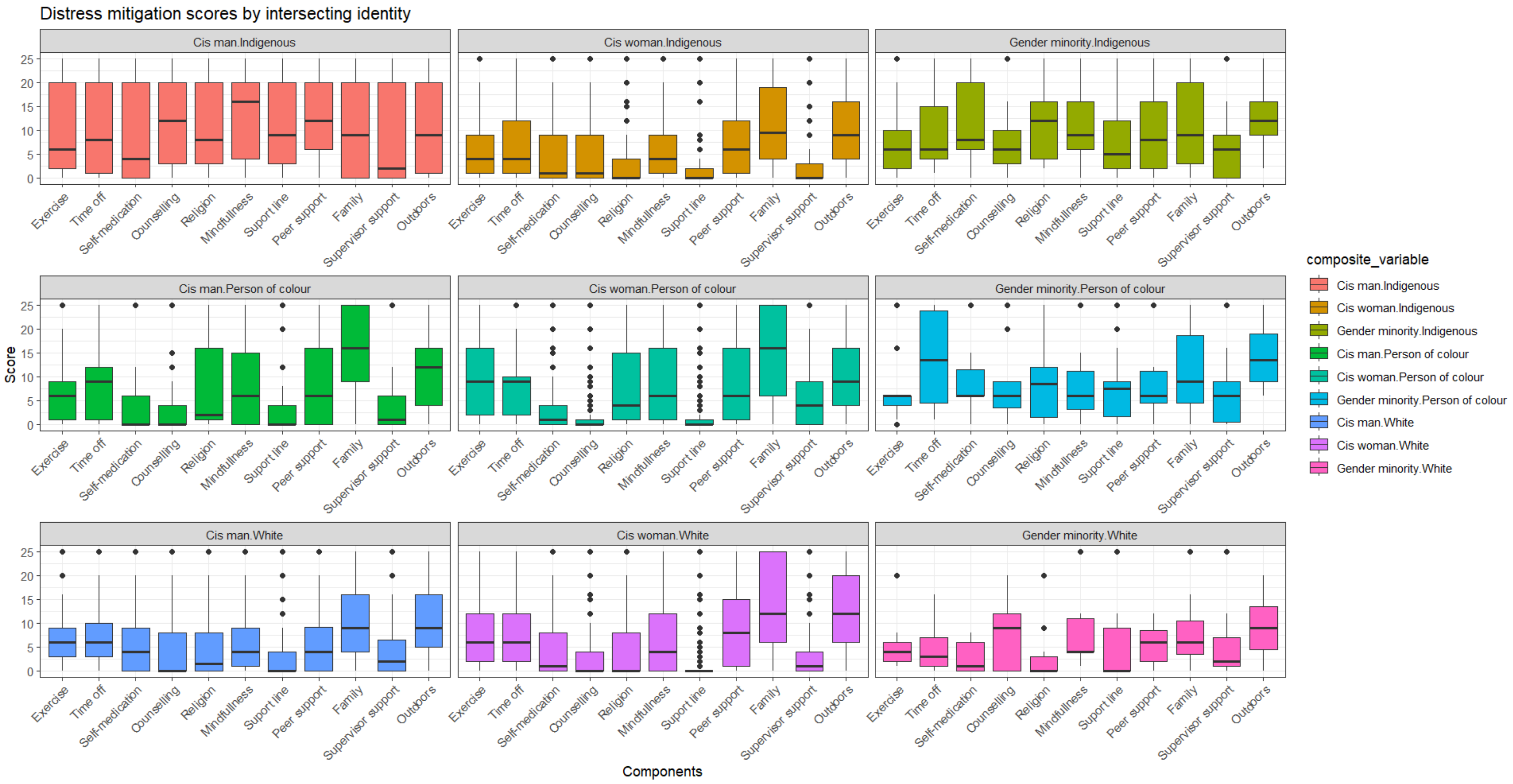
Distress mitigation scores by intersecting identity
Cis man.Indigenous
Cis woman.Indigenous
Gender minority.Indigenous
Cis man.Person of colour
Cis woman.Person of colour
Gender minority.Person of colour
Cis man.White
Cis woman.White
Gender minority.White
Score
Components
Exercise
Time off
Self-medication
Counselling
Religion
Mindfullness
Suport line
Peer support
Family
Supervisor support
Outdoors
composite_variable
Cis man.Indigenous
Cis woman.Indigenous
Gender minority.Indigenous
Cis man.Person of colour
Cis woman.Person of colour
Gender minority.Person of colour
Cis man.White
Cis woman.White
Gender minority.White

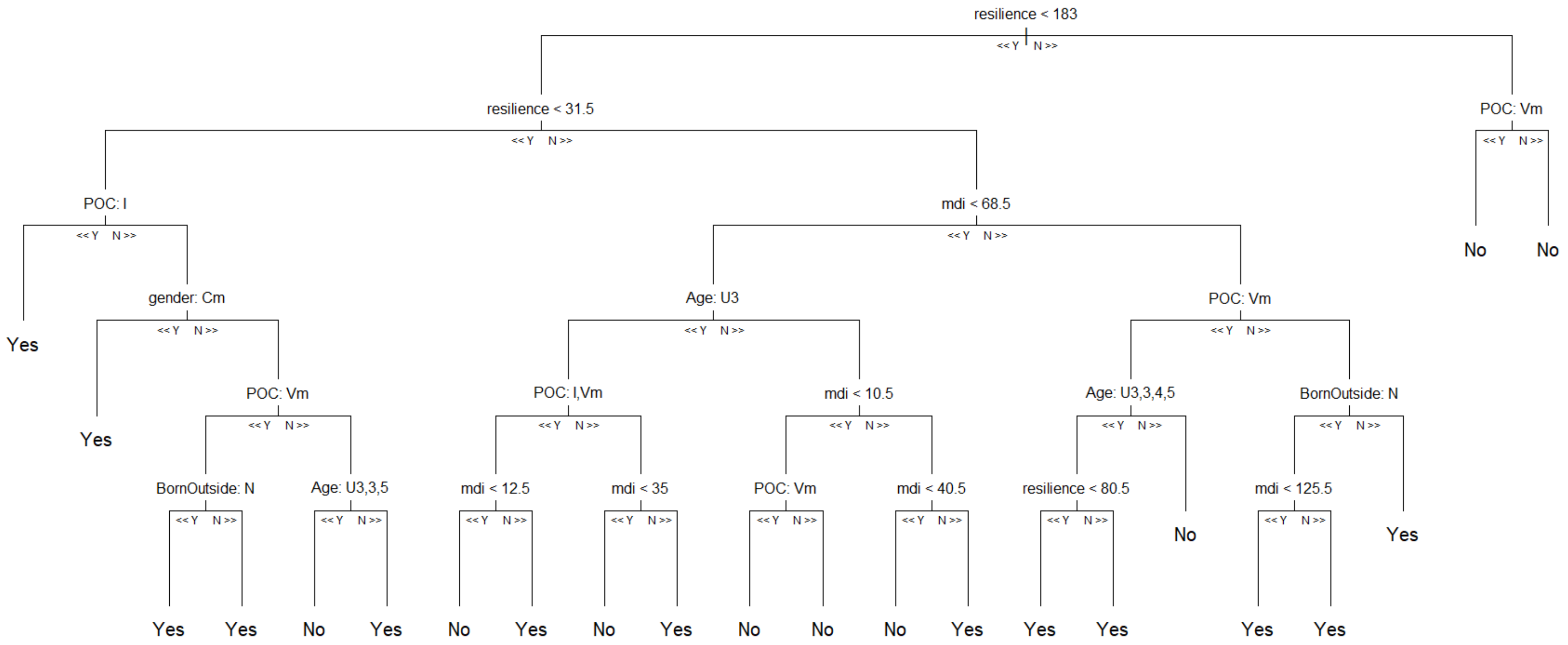
resilience < 183
resilience < 31.5
POC: Vm
POC: I
mdi < 68.5
No
No
Yes
gender: Cm
Age: U3
POC: Vm
Yes
POC: Vm
POC: I,Vm
mdi < 10.5
Age: U3,3,4,5
BornOutside: N
BornOutside: N
Age: U3,3,5
mdi < 12.5
mdi < 35
POC: Vm
mdi < 40.5
resilience < 80.5
No
mdi < 125.5
Yes
Yes
Yes
No
Yes
No
Yes
No
Yes
No
No
No
Yes
Yes
Yes
Yes
Yes

Supplementary data

Kruskal-Wallis test results and Dunn test (if relevant) for moral distress components by intersecting groups of gender and racialized experiences.

[[1]]

	variable	test	statistic	p.value
Kruskal-Wallis chi-squared	Unqualified	Kruskal-Wallis	22.01144	0.00489471

[[2]]

	variable	test	comparison	Z	p.value
1	Unqualified	Dunn	GM of colour - Ind. men	-0.72137728	1.000000000
2	Unqualified	Dunn	GM of colour - Ind. women	0.62652072	1.000000000
3	Unqualified	Dunn	Ind. men - Ind. women	1.99469185	0.829376977
4	Unqualified	Dunn	GM of colour - Men of colour	0.77480103	1.000000000
5	Unqualified	Dunn	Ind. men - Men of colour	1.86856748	1.000000000
6	Unqualified	Dunn	Ind. women - Men of colour	0.34306618	1.000000000
7	Unqualified	Dunn	GM of colour - TwoS	0.56338987	1.000000000
8	Unqualified	Dunn	Ind. men - TwoS	1.34274995	1.000000000
9	Unqualified	Dunn	Ind. women - TwoS	0.13413663	1.000000000
10	Unqualified	Dunn	Men of colour - TwoS	-0.09691144	1.000000000
11	Unqualified	Dunn	GM of colour - White GM	1.55693985	1.000000000
12	Unqualified	Dunn	Ind. men - White GM	2.39923901	0.295725364
13	Unqualified	Dunn	Ind. women - White GM	1.39463443	1.000000000
14	Unqualified	Dunn	Men of colour - White GM	1.06600032	1.000000000
15	Unqualified	Dunn	TwoS - White GM	1.00179425	1.000000000
16	Unqualified	Dunn	GM of colour - White men	1.91722801	0.993761469
17	Unqualified	Dunn	Ind. men - White men	3.75469080	0.003124001
18	Unqualified	Dunn	Ind. women - White men	2.47778671	0.237960264
19	Unqualified	Dunn	Men of colour - White men	1.37415206	1.000000000
20	Unqualified	Dunn	TwoS - White men	1.08957256	1.000000000
21	Unqualified	Dunn	White GM - White men	-0.28796621	1.000000000
22	Unqualified	Dunn	GM of colour - White women	1.50302197	1.000000000
**23**	**Unqualified**	**Dunn**	**Ind. men - White women**	**3.50389173**	**0.008253211**
24	Unqualified	Dunn	Ind. women - White women	2.05256594	0.722064494
25	Unqualified	Dunn	Men of colour - White women	0.78868243	1.000000000
26	Unqualified	Dunn	TwoS - White women	0.64221767	1.000000000
27	Unqualified	Dunn	White GM - White women	-0.76008669	1.000000000
28	Unqualified	Dunn	White men - White women	-1.28550937	1.000000000
29	Unqualified	Dunn	GM of colour - Women of colour	1.43944941	1.000000000
**30**	**Unqualified**	**Dunn**	**Ind. men - Women of colour**	**3.34178017**	**0.014983733**
31	Unqualified	Dunn	Ind. women - Women of colour	1.80479627	1.000000000
32	Unqualified	Dunn	Men of colour - Women of colour	0.70792617	1.000000000
33	Unqualified	Dunn	TwoS - Women of colour	0.59354962	1.000000000
34	Unqualified	Dunn	White GM - Women of colour	-0.79003188	1.000000000
35	Unqualified	Dunn	White men - Women of colour	-1.29927211	1.000000000
36	Unqualified	Dunn	White women - Women of colour	-0.17855022	1.000000000

[[3]]

	variable	test	statistic	p.value
Kruskal-Wallis chi-squared	Discontinuity	Kruskal-Wallis	22.29905	0.004390979

[[4]]

	variable	test	comparison	Z	p.value
1	Discontinuity	Dunn	GM of colour - Ind. men	-0.92644009	1.000000000
2	Discontinuity	Dunn	GM of colour - Ind. women	-0.86316489	1.000000000
3	Discontinuity	Dunn	Ind. men - Ind. women	0.24289561	1.000000000
4	Discontinuity	Dunn	GM of colour - Men of colour	0.49672366	1.000000000

5	Discontinuity	Dunn	Ind. men - Men of colour	1.77171982	1.000000000
6	Discontinuity	Dunn	Ind. women - Men of colour	1.90178527	1.000000000
7	Discontinuity	Dunn	GM of colour - TwoS	0.12925105	1.000000000
8	Discontinuity	Dunn	Ind. men - TwoS	1.03394367	1.000000000
9	Discontinuity	Dunn	Ind. women - TwoS	0.98045761	1.000000000
10	Discontinuity	Dunn	Men of colour - TwoS	-0.32702080	1.000000000
11	Discontinuity	Dunn	GM of colour - White GM	0.15079232	1.000000000
12	Discontinuity	Dunn	Ind. men - White GM	0.97942739	1.000000000
13	Discontinuity	Dunn	Ind. women - White GM	0.91728069	1.000000000
14	Discontinuity	Dunn	Men of colour - White GM	-0.26616605	1.000000000
15	Discontinuity	Dunn	TwoS - White GM	0.02918879	1.000000000
16	Discontinuity	Dunn	GM of colour - White men	1.01208722	1.000000000
17	Discontinuity	Dunn	Ind. men - White men	2.81972625	0.086516340
**18**	**Discontinuity**	**Dunn**	**Ind. women - White men**	**3.57447879**	**0.006316673**
19	Discontinuity	Dunn	Men of colour - White men	0.59400526	1.000000000
20	Discontinuity	Dunn	TwoS - White men	0.79013908	1.000000000
21	Discontinuity	Dunn	White GM - White men	0.67329948	1.000000000
22	Discontinuity	Dunn	GM of colour - White women	-0.29631982	1.000000000
23	Discontinuity	Dunn	Ind. men - White women	1.19709884	1.000000000
24	Discontinuity	Dunn	Ind. women - White women	1.52365740	1.000000000
25	Discontinuity	Dunn	Men of colour - White women	-1.26027776	1.000000000
26	Discontinuity	Dunn	TwoS - White women	-0.45307242	1.000000000
27	Discontinuity	Dunn	White GM - White women	-0.44104986	1.000000000
**28**	**Discontinuity**	**Dunn**	**White men - White women**	**-3.22299157**	**0.022834672**
29	Discontinuity	Dunn	GM of colour - Women of colour	0.30202539	1.000000000
30	Discontinuity	Dunn	Ind. men - Women of colour	2.03224330	0.758322565
31	Discontinuity	Dunn	Ind. women - Women of colour	2.78724979	0.095683477
32	Discontinuity	Dunn	Men of colour - Women of colour	-0.40566142	1.000000000
33	Discontinuity	Dunn	TwoS - Women of colour	0.10971191	1.000000000
34	Discontinuity	Dunn	White GM - Women of colour	0.05923786	1.000000000
35	Discontinuity	Dunn	White men - Women of colour	-1.69837668	1.000000000
36	Discontinuity	Dunn	White women - Women of colour	2.40798282	0.288736843

[[5]]

	variable	test	statistic	p.value
Kruskal-Wallis chi-squared	IncompetentColleagues	Kruskal-Wallis	24.46923	0.001911178

[[6]]

	variable	test	comparison	Z	p.value
1	IncompetentColleagues	Dunn	GM of colour - Ind. men	-0.95833932	1.000000000
2	IncompetentColleagues	Dunn	GM of colour - Ind. women	-0.86672264	1.000000000
3	IncompetentColleagues	Dunn	Ind. men - Ind. women	0.28763467	1.000000000
4	IncompetentColleagues	Dunn	GM of colour - Men of colour	0.34009652	1.000000000
5	IncompetentColleagues	Dunn	Ind. men - Men of colour	1.61377972	1.000000000
6	IncompetentColleagues	Dunn	Ind. women - Men of colour	1.66875479	1.000000000
7	IncompetentColleagues	Dunn	GM of colour - TwoS	0.55394755	1.000000000
8	IncompetentColleagues	Dunn	Ind. men - TwoS	1.55782634	1.000000000
9	IncompetentColleagues	Dunn	Ind. women - TwoS	1.53037797	1.000000000
10	IncompetentColleagues	Dunn	Men of colour - TwoS	0.30767921	1.000000000
11	IncompetentColleagues	Dunn	GM of colour - White GM	0.21471796	1.000000000
12	IncompetentColleagues	Dunn	Ind. men - White GM	1.07992556	1.000000000
13	IncompetentColleagues	Dunn	Ind. women - White GM	0.99933483	1.000000000
14	IncompetentColleagues	Dunn	Men of colour - White GM	-0.05731771	1.000000000
15	IncompetentColleagues	Dunn	TwoS - White GM	-0.29441813	1.000000000
16	IncompetentColleagues	Dunn	GM of colour - White men	0.41007477	1.000000000
17	IncompetentColleagues	Dunn	Ind. men - White men	2.03746567	0.748861172
18	IncompetentColleagues	Dunn	Ind. women - White men	2.42973825	0.271975146
19	IncompetentColleagues	Dunn	Men of colour - White men	0.03269789	1.000000000
20	IncompetentColleagues	Dunn	TwoS - White men	-0.32104490	1.000000000
21	IncompetentColleagues	Dunn	White GM - White men	0.08388500	1.000000000
22	IncompetentColleagues	Dunn	GM of colour - White women	-0.39919432	1.000000000
23	IncompetentColleagues	Dunn	Ind. men - White women	1.10085286	1.000000000

	variable	test	comparison	Z	p.value
24	IncompetentColleagues	Dunn	Ind. women - White women	1.28064705	1.000000000
25	IncompetentColleagues	Dunn	Men of colour - White women	-1.13803761	1.000000000
26	IncompetentColleagues	Dunn	TwoS - White women	-1.12632781	1.000000000
27	IncompetentColleagues	Dunn	White GM - White women	-0.60911240	1.000000000
28	IncompetentColleagues	Dunn	White men - White women	-1.95760902	0.904966347
29	IncompetentColleagues	Dunn	GM of colour - Women of colour	0.54336975	1.000000000
30	IncompetentColleagues	Dunn	Ind. men - Women of colour	2.43961227	0.264654595
**31**	**IncompetentColleagues**	**Dunn**	**Ind. women - Women of colour**	**3.35284179**	**0.014397554**
32	IncompetentColleagues	Dunn	Men of colour - Women of colour	0.18825098	1.000000000
33	IncompetentColleagues	Dunn	TwoS - Women of colour	-0.23425656	1.000000000
34	IncompetentColleagues	Dunn	White GM - Women of colour	0.17894227	1.000000000
35	IncompetentColleagues	Dunn	White men - Women of colour	0.24286530	1.000000000
**36**	**IncompetentColleagues**	**Dunn**	**White women - Women of colour**	**3.79680887**	**0.002638273**

[[7]]

	variable	test	statistic	p.value
Kruskal-Wallis chi-squared	Miscommunication	Kruskal-Wallis	19.03688	0.01466365

[[8]]

	variable	test	comparison	Z	p.value
1	Miscommunication	Dunn	GM of colour - Ind. men	-0.348308704	1.000000000
2	Miscommunication	Dunn	GM of colour - Ind. women	-0.347509285	1.000000000
3	Miscommunication	Dunn	Ind. men - Ind. women	0.059356052	1.000000000
4	Miscommunication	Dunn	GM of colour - Men of colour	-0.002325154	1.000000000
5	Miscommunication	Dunn	Ind. men - Men of colour	0.427820963	1.000000000
6	Miscommunication	Dunn	Ind. women - Men of colour	0.458555304	1.000000000
7	Miscommunication	Dunn	GM of colour - TwoS	-0.774532655	1.000000000
8	Miscommunication	Dunn	Ind. men - TwoS	-0.567660295	1.000000000
9	Miscommunication	Dunn	Ind. women - TwoS	-0.669024865	1.000000000
10	Miscommunication	Dunn	Men of colour - TwoS	-0.882356865	1.000000000
11	Miscommunication	Dunn	GM of colour - White GM	-0.360590051	1.000000000
12	Miscommunication	Dunn	Ind. men - White GM	-0.106180693	1.000000000
13	Miscommunication	Dunn	Ind. women - White GM	-0.151625150	1.000000000
14	Miscommunication	Dunn	Men of colour - White GM	-0.402051486	1.000000000
15	Miscommunication	Dunn	TwoS - White GM	0.352970627	1.000000000
16	Miscommunication	Dunn	GM of colour - White men	0.310576705	1.000000000
17	Miscommunication	Dunn	Ind. men - White men	0.963557868	1.000000000
18	Miscommunication	Dunn	Ind. women - White men	1.253758627	1.000000000
19	Miscommunication	Dunn	Men of colour - White men	0.414106372	1.000000000
20	Miscommunication	Dunn	TwoS - White men	1.283293575	1.000000000
21	Miscommunication	Dunn	White GM - White men	0.706969962	1.000000000
22	Miscommunication	Dunn	GM of colour - White women	-0.069732486	1.000000000
23	Miscommunication	Dunn	Ind. men - White women	0.511882939	1.000000000
24	Miscommunication	Dunn	Ind. women - White women	0.734975676	1.000000000
25	Miscommunication	Dunn	Men of colour - White women	-0.093782862	1.000000000
26	Miscommunication	Dunn	TwoS - White women	0.986838287	1.000000000
27	Miscommunication	Dunn	White GM - White women	0.407503765	1.000000000
28	Miscommunication	Dunn	White men - White women	-0.939536728	1.000000000
29	Miscommunication	Dunn	GM of colour - Women of colour	0.862478010	1.000000000
30	Miscommunication	Dunn	Ind. men - Women of colour	1.858182320	1.000000000
31	Miscommunication	Dunn	Ind. women - Women of colour	2.831959771	0.083274604
32	Miscommunication	Dunn	Men of colour - Women of colour	1.197970445	1.000000000
33	Miscommunication	Dunn	TwoS - Women of colour	1.849116537	1.000000000
34	Miscommunication	Dunn	White GM - Women of colour	1.181311979	1.000000000
35	Miscommunication	Dunn	White men - Women of colour	1.181184743	1.000000000
**36**	**Miscommunication**	**Dunn**	**White women - Women of colour**	**3.772177313**	**0.002912925**

[[9]]

	variable	test	statistic	p.value
Kruskal-Wallis chi-squared	Overload	Kruskal-Wallis	34.83779	0.00002862007

[[10]]

	variable	test	comparison	Z	p.value
1	Overload	Dunn	GM of colour - Ind. men	-0.82890512	1.00000000000
2	Overload	Dunn	GM of colour - Ind. women	-1.06196640	1.00000000000
3	Overload	Dunn	Ind. men - Ind. women	-0.18542844	1.00000000000
4	Overload	Dunn	GM of colour - Men of colour	-0.19062957	1.00000000000
5	Overload	Dunn	Ind. men - Men of colour	0.78486059	1.00000000000
6	Overload	Dunn	Ind. women - Men of colour	1.12272508	1.00000000000
7	Overload	Dunn	GM of colour - TwoS	-0.18309046	1.00000000000
8	Overload	Dunn	Ind. men - TwoS	0.57799319	1.00000000000
9	Overload	Dunn	Ind. women - TwoS	0.76599360	1.00000000000
10	Overload	Dunn	Men of colour - TwoS	-0.02695119	1.00000000000
11	Overload	Dunn	GM of colour - White GM	-0.32996057	1.00000000000
12	Overload	Dunn	Ind. men - White GM	0.34779227	1.00000000000
13	Overload	Dunn	Ind. women - White GM	0.49116931	1.00000000000
14	Overload	Dunn	Men of colour - White GM	-0.20276540	1.00000000000
15	Overload	Dunn	TwoS - White GM	-0.15445816	1.00000000000
16	Overload	Dunn	GM of colour - White men	0.53617299	1.00000000000
17	Overload	Dunn	Ind. men - White men	2.01286791	0.79431348183
**18**	**Overload**	**Dunn**	**Ind. women - White men**	**3.04157895**	**0.04236135125**
19	Overload	Dunn	Men of colour - White men	0.99446001	1.00000000000
20	Overload	Dunn	TwoS - White men	0.74017769	1.00000000000
21	Overload	Dunn	White GM - White men	0.86071754	1.00000000000
22	Overload	Dunn	GM of colour - White women	-0.95367896	1.00000000000
23	Overload	Dunn	Ind. men - White women	0.04969942	1.00000000000
24	Overload	Dunn	Ind. women - White women	0.42986545	1.00000000000
25	Overload	Dunn	Men of colour - White women	-1.01241993	1.00000000000
26	Overload	Dunn	TwoS - White women	-0.64437977	1.00000000000
27	Overload	Dunn	White GM - White women	-0.36704249	1.00000000000
**28**	**Overload**	**Dunn**	**White men - White women**	**-3.56759552**	**0.00648489716**
29	Overload	Dunn	GM of colour - Women of colour	0.26059207	1.00000000000
30	Overload	Dunn	Ind. men - Women of colour	1.80421217	1.00000000000
**31**	**Overload**	**Dunn**	**Ind. women - Women of colour**	**3.17304573**	**0.02715277729**
32	Overload	Dunn	Men of colour - Women of colour	0.67695445	1.00000000000
33	Overload	Dunn	TwoS - Women of colour	0.49030343	1.00000000000
34	Overload	Dunn	White GM - Women of colour	0.63965558	1.00000000000
35	Overload	Dunn	White men - Women of colour	-0.67730304	1.00000000000
**36**	**Overload**	**Dunn**	**White women - Women of colour**	**4.86821110**	**0.00002027034**

[[11]]

	variable	test	statistic	p.value
Kruskal-Wallis chi-squared	Resources	Kruskal-Wallis	25.13211	0.001476406

[[12]]

	variable	test	comparison	Z	p.value
1	Resources	Dunn	GM of colour - Ind. men	-0.27985923	1.000000000
2	Resources	Dunn	GM of colour - Ind. women	0.30049221	1.000000000
3	Resources	Dunn	Ind. men - Ind. women	0.85369531	1.000000000
4	Resources	Dunn	GM of colour - Men of colour	0.19670891	1.000000000
5	Resources	Dunn	Ind. men - Men of colour	0.59400300	1.000000000
6	Resources	Dunn	Ind. women - Men of colour	-0.10096039	1.000000000
7	Resources	Dunn	GM of colour - TwoS	-0.30520109	1.000000000
8	Resources	Dunn	Ind. men - TwoS	-0.08764294	1.000000000
9	Resources	Dunn	Ind. women - TwoS	-0.67619711	1.000000000
10	Resources	Dunn	Men of colour - TwoS	-0.53652608	1.000000000
11	Resources	Dunn	GM of colour - White GM	0.05714077	1.000000000
12	Resources	Dunn	Ind. men - White GM	0.30904168	1.000000000
13	Resources	Dunn	Ind. women - White GM	-0.18377054	1.000000000
14	Resources	Dunn	Men of colour - White GM	-0.10829068	1.000000000
15	Resources	Dunn	TwoS - White GM	0.33275983	1.000000000
16	Resources	Dunn	GM of colour - White men	0.99570557	1.000000000
17	Resources	Dunn	Ind. men - White men	1.80446967	1.000000000
18	Resources	Dunn	Ind. women - White men	1.33407877	1.000000000

	variable	test	comparison	Z	p.value
19	Resources	Dunn	Men of colour - White men	1.02177773	1.000000000
20	Resources	Dunn	TwoS - White men	1.33005665	1.000000000
21	Resources	Dunn	White GM - White men	0.77457156	1.000000000
22	Resources	Dunn	GM of colour - White women	0.62833981	1.000000000
23	Resources	Dunn	Ind. men - White women	1.42644684	1.000000000
24	Resources	Dunn	Ind. women - White women	0.75751006	1.000000000
25	Resources	Dunn	Men of colour - White women	0.54607859	1.000000000
26	Resources	Dunn	TwoS - White women	1.00301392	1.000000000
27	Resources	Dunn	White GM - White women	0.44870499	1.000000000
28	Resources	Dunn	White men - White women	-1.02310079	1.000000000
29	Resources	Dunn	GM of colour - Women of colour	1.61118558	1.000000000
30	Resources	Dunn	Ind. men - Women of colour	2.83444178	0.082630478
31	Resources	Dunn	Ind. women - Women of colour	2.99063841	0.050111087
32	Resources	Dunn	Men of colour - Women of colour	1.90446804	1.000000000
33	Resources	Dunn	TwoS - Women of colour	1.92109233	0.984961230
34	Resources	Dunn	White GM - Women of colour	1.27197067	1.000000000
35	Resources	Dunn	White men - Women of colour	1.23505879	1.000000000
**36**	**Resources**	**Dunn**	**White women - Women of colour**	**4.01393689**	**0.001074858**

[[13]]

	variable	test	statistic	p.value
Kruskal-Wallis chi-squared	AdminSupport	Kruskal-Wallis	30.10145	0.0002028249

[[14]]

	variable	test	comparison	Z	p.value
1	AdminSupport	Dunn	GM of colour - Ind. men	-0.21240858	1.00000000000
2	AdminSupport	Dunn	GM of colour - Ind. women	-0.16841659	1.00000000000
3	AdminSupport	Dunn	Ind. men - Ind. women	0.09668387	1.00000000000
4	AdminSupport	Dunn	GM of colour - Men of colour	-0.27562668	1.00000000000
5	AdminSupport	Dunn	Ind. men - Men of colour	-0.08467470	1.00000000000
6	AdminSupport	Dunn	Ind. women - Men of colour	-0.19445905	1.00000000000
7	AdminSupport	Dunn	GM of colour - TwoS	0.45518639	1.00000000000
8	AdminSupport	Dunn	Ind. men - TwoS	0.73150366	1.00000000000
9	AdminSupport	Dunn	Ind. women - TwoS	0.74465053	1.00000000000
10	AdminSupport	Dunn	Men of colour - TwoS	0.78322990	1.00000000000
11	AdminSupport	Dunn	GM of colour - White GM	0.19687451	1.00000000000
12	AdminSupport	Dunn	Ind. men - White GM	0.40907888	1.00000000000
13	AdminSupport	Dunn	Ind. women - White GM	0.38602475	1.00000000000
14	AdminSupport	Dunn	Men of colour - White GM	0.46208302	1.00000000000
15	AdminSupport	Dunn	TwoS - White GM	-0.22205967	1.00000000000
16	AdminSupport	Dunn	GM of colour - White men	1.02452867	1.00000000000
17	AdminSupport	Dunn	Ind. men - White men	1.74071644	1.00000000000
18	AdminSupport	Dunn	Ind. women - White men	2.27937813	0.40760274494
19	AdminSupport	Dunn	Men of colour - White men	1.76745863	1.00000000000
20	AdminSupport	Dunn	TwoS - White men	0.38522440	1.00000000000
21	AdminSupport	Dunn	White GM - White men	0.62718450	1.00000000000
22	AdminSupport	Dunn	GM of colour - White women	0.13313220	1.00000000000
23	AdminSupport	Dunn	Ind. men - White women	0.57272194	1.00000000000
24	AdminSupport	Dunn	Ind. women - White women	0.76556439	1.00000000000
25	AdminSupport	Dunn	Men of colour - White women	0.65545468	1.00000000000
26	AdminSupport	Dunn	TwoS - White women	-0.49365982	1.00000000000
27	AdminSupport	Dunn	White GM - White women	-0.14344567	1.00000000000
28	AdminSupport	Dunn	White men - White women	-2.25140961	0.43847291182
29	AdminSupport	Dunn	GM of colour - Women of colour	1.29601066	1.00000000000
30	AdminSupport	Dunn	Ind. men - Women of colour	2.25815791	0.43084469860
31	AdminSupport	Dunn	Ind. women - Women of colour	3.39878074	0.01218365199
32	AdminSupport	Dunn	Men of colour - Women of colour	2.25147593	0.43839737914
33	AdminSupport	Dunn	TwoS - Women of colour	0.60432009	1.00000000000
34	AdminSupport	Dunn	White GM - Women of colour	0.83009704	1.00000000000
35	AdminSupport	Dunn	White men - Women of colour	0.47067782	1.00000000000
**36**	**AdminSupport**	**Dunn**	**White women - Women of colour**	**4.71713377**	**0.00004305428**

[[15]]

	variable	test	statistic	p.value
Kruskal-Wallis chi-squared	TimeForPatient	Kruskal-Wallis	59.11847	0.0000000006938841

[[16]]

	variable	test	comparison	Z	p.value
1	TimeForPatient	Dunn	GM of colour - Ind. men	-1.22953074	1.0000000000000000
2	TimeForPatient	Dunn	GM of colour - Ind. women	-0.57010849	1.0000000000000000
3	TimeForPatient	Dunn	Ind. men - Ind. women	1.12243727	1.0000000000000000
4	TimeForPatient	Dunn	GM of colour - Men of colour	-0.06444255	1.0000000000000000
5	TimeForPatient	Dunn	Ind. men - Men of colour	1.43933929	1.0000000000000000
6	TimeForPatient	Dunn	Ind. women - Men of colour	0.66025194	1.0000000000000000
7	TimeForPatient	Dunn	GM of colour - TwoS	-0.22230406	1.0000000000000000
8	TimeForPatient	Dunn	Ind. men - TwoS	0.91460359	1.0000000000000000
9	TimeForPatient	Dunn	Ind. women - TwoS	0.25161832	1.0000000000000000
10	TimeForPatient	Dunn	Men of colour - TwoS	-0.19231214	1.0000000000000000
11	TimeForPatient	Dunn	GM of colour - White GM	-0.59694734	1.0000000000000000
12	TimeForPatient	Dunn	Ind. men - White GM	0.39365760	1.0000000000000000
13	TimeForPatient	Dunn	Ind. women - White GM	-0.25540066	1.0000000000000000
14	TimeForPatient	Dunn	Men of colour - White GM	-0.61250363	1.0000000000000000
15	TimeForPatient	Dunn	TwoS - White GM	-0.37838145	1.0000000000000000
16	TimeForPatient	Dunn	GM of colour - White men	0.76105282	1.0000000000000000
17	TimeForPatient	Dunn	Ind. men - White men	2.93841672	0.0593807901342927
18	TimeForPatient	Dunn	Ind. women - White men	2.53798165	0.2006888969167442
19	TimeForPatient	Dunn	Men of colour - White men	1.10274873	1.0000000000000000
20	TimeForPatient	Dunn	TwoS - White men	1.00258324	1.0000000000000000
21	TimeForPatient	Dunn	White GM - White men	1.37986254	1.0000000000000000
22	TimeForPatient	Dunn	GM of colour - White women	-0.37587576	1.0000000000000000
23	TimeForPatient	Dunn	Ind. men - White women	1.61452810	1.0000000000000000
24	TimeForPatient	Dunn	Ind. women - White women	0.56453532	1.0000000000000000
25	TimeForPatient	Dunn	Men of colour - White women	-0.41721295	1.0000000000000000
26	TimeForPatient	Dunn	TwoS - White women	-0.05001477	1.0000000000000000
27	TimeForPatient	Dunn	White GM - White women	0.45805817	1.0000000000000000
28	TimeForPatient	Dunn	White men - White women	-2.78092750	0.0975669134637424
29	TimeForPatient	Dunn	GM of colour - Women of colour	1.30826477	1.0000000000000000
**30**	**TimeForPatient**	**Dunn**	**Ind. men - Women of colour**	**4.02279662**	**0.0010352008143406**
**31**	**TimeForPatient**	**Dunn**	**Ind. women - Women of colour**	**4.39979469**	**0.0001950360182246**
32	TimeForPatient	Dunn	Men of colour - Women of colour	1.91819132	0.9915616067277296
33	TimeForPatient	Dunn	TwoS - Women of colour	1.52563950	1.0000000000000000
34	TimeForPatient	Dunn	White GM - Women of colour	1.85583106	1.0000000000000000
35	TimeForPatient	Dunn	White men - Women of colour	1.11379119	1.0000000000000000
**36**	**TimeForPatient**	**Dunn**	**White women - Women of colour**	**6.80172655**	**0.0000000001860708**

[[17]]

	variable	test	statistic	p.value
Kruskal-Wallis chi-squared	TimeForSelf	Kruskal-Wallis	37.34767	0.000009932029

[[18]]

	variable	test	comparison	Z	p.value
1	TimeForSelf	Dunn	GM of colour - Ind. men	-0.65523127	1.0000000000
2	TimeForSelf	Dunn	GM of colour - Ind. women	-0.70882544	1.0000000000
3	TimeForSelf	Dunn	Ind. men - Ind. women	0.03505337	1.0000000000
4	TimeForSelf	Dunn	GM of colour - Men of colour	0.12923129	1.0000000000
5	TimeForSelf	Dunn	Ind. men - Men of colour	0.97318450	1.0000000000
6	TimeForSelf	Dunn	Ind. women - Men of colour	1.13870298	1.0000000000
7	TimeForSelf	Dunn	GM of colour - TwoS	0.20958557	1.0000000000
8	TimeForSelf	Dunn	Ind. men - TwoS	0.86856723	1.0000000000
9	TimeForSelf	Dunn	Ind. women - TwoS	0.93827498	1.0000000000
10	TimeForSelf	Dunn	Men of colour - TwoS	0.11587882	1.0000000000
11	TimeForSelf	Dunn	GM of colour - White GM	1.09054148	1.0000000000
12	TimeForSelf	Dunn	Ind. men - White GM	1.81129936	1.0000000000
13	TimeForSelf	Dunn	Ind. women - White GM	1.94857047	0.9242413522

14	TimeForSelf	Dunn	Men of colour - White GM	1.10888318	1.0000000000
15	TimeForSelf	Dunn	TwoS - White GM	0.86976193	1.0000000000
16	TimeForSelf	Dunn	GM of colour - White men	1.38964177	1.0000000000
17	TimeForSelf	Dunn	Ind. men - White men	2.92467463	0.0620669777
**18**	**TimeForSelf**	**Dunn**	**Ind. women - White men**	**4.00364797**	**0.0011227169**
19	TimeForSelf	Dunn	Men of colour - White men	1.64369668	1.0000000000
20	TimeForSelf	Dunn	TwoS - White men	1.04383889	1.0000000000
21	TimeForSelf	Dunn	White GM - White men	-0.16204419	1.0000000000
22	TimeForSelf	Dunn	GM of colour - White women	-0.24067961	1.0000000000
23	TimeForSelf	Dunn	Ind. men - White women	0.80051644	1.0000000000
24	TimeForSelf	Dunn	Ind. women - White women	1.25773188	1.0000000000
25	TimeForSelf	Dunn	Men of colour - White women	-0.55716886	1.0000000000
26	TimeForSelf	Dunn	TwoS - White women	-0.51009497	1.0000000000
27	TimeForSelf	Dunn	White GM - White women	-1.60790959	1.0000000000
**28**	**TimeForSelf**	**Dunn**	**White men - White women**	**-4.03730989**	**0.0009732173**
29	TimeForSelf	Dunn	GM of colour - Women of colour	0.78178815	1.0000000000
30	TimeForSelf	Dunn	Ind. men - Women of colour	2.26738793	0.4205976851
**31**	**TimeForSelf**	**Dunn**	**Ind. women - Women of colour**	**3.52070325**	**0.0077472719**
32	TimeForSelf	Dunn	Men of colour - Women of colour	0.86806665	1.0000000000
33	TimeForSelf	Dunn	TwoS - Women of colour	0.45182600	1.0000000000
34	TimeForSelf	Dunn	White GM - Women of colour	-0.74242383	1.0000000000
35	TimeForSelf	Dunn	White men - Women of colour	-1.52014151	1.0000000000
**36**	**TimeForSelf**	**Dunn**	**White women - Women of colour**	**4.12877787**	**0.0006564537**

Kruskal-Wallis test results and Dunn test (if relevant) for coping strategies by intersecting groups of gender and racialized experiences.

[[1]]

	variable	test	statistic	p.value
Kruskal-Wallis chi-squared	Exercise	Kruskal-Wallis	9.597844	0.2943934

[[2]]

	variable	test	statistic	p.value
Kruskal-Wallis chi-squared	Time off	Kruskal-Wallis	16.7766	0.03252129

[[3]]

	variable	test	comparison	Z	p.value
1	Time off	Dunn	GM of colour - Ind. men	1.0684194	1.0000000
2	Time off	Dunn	GM of colour - Ind. women	2.6186086	0.1589205
3	Time off	Dunn	Ind. men - Ind. women	1.9766574	0.8654466
4	Time off	Dunn	GM of colour - Men of colour	1.2971491	1.0000000
5	Time off	Dunn	Ind. men - Men of colour	0.3194760	1.0000000
6	Time off	Dunn	Ind. women - Men of colour	-1.4840633	1.0000000
7	Time off	Dunn	GM of colour - TwoS	0.6522722	1.0000000
8	Time off	Dunn	Ind. men - TwoS	-0.2613354	1.0000000
9	Time off	Dunn	Ind. women - TwoS	-1.6303679	1.0000000
10	Time off	Dunn	Men of colour - TwoS	-0.4987253	1.0000000
11	Time off	Dunn	GM of colour - White GM	2.6742989	0.1347942
12	Time off	Dunn	Ind. men - White GM	2.1086065	0.6296141
13	Time off	Dunn	Ind. women - White GM	1.0895333	1.0000000
14	Time off	Dunn	Men of colour - White GM	1.8468534	1.0000000
15	Time off	Dunn	TwoS - White GM	2.0072600	0.8049957
16	Time off	Dunn	GM of colour - White men	1.9557995	0.9087980
17	Time off	Dunn	Ind. men - White men	1.0602345	1.0000000
18	Time off	Dunn	Ind. women - White men	-1.2301806	1.0000000
19	Time off	Dunn	Men of colour - White men	0.6247436	1.0000000
20	Time off	Dunn	TwoS - White men	1.0123598	1.0000000
21	Time off	Dunn	White GM - White men	-1.6293552	1.0000000
22	Time off	Dunn	GM of colour - White women	2.2705826	0.4171006
23	Time off	Dunn	Ind. men - White women	1.4806110	1.0000000
24	Time off	Dunn	Ind. women - White women	-1.2580843	1.0000000
25	Time off	Dunn	Men of colour - White women	0.9495328	1.0000000
26	Time off	Dunn	TwoS - White women	1.2403204	1.0000000
27	Time off	Dunn	White GM - White women	-1.5641338	1.0000000
28	Time off	Dunn	White men - White women	0.4102773	1.0000000
29	Time off	Dunn	GM of colour - Women of colour	1.8220068	1.0000000
30	Time off	Dunn	Ind. men - Women of colour	0.8271617	1.0000000
31	Time off	Dunn	Ind. women - Women of colour	-2.1334663	0.5919569
32	Time off	Dunn	Men of colour - Women of colour	0.3548635	1.0000000
33	Time off	Dunn	TwoS - Women of colour	0.8331912	1.0000000
34	Time off	Dunn	White GM - Women of colour	-1.8995241	1.0000000
35	Time off	Dunn	White men - Women of colour	-0.5422077	1.0000000
36	Time off	Dunn	White women - Women of colour	-1.6903963	1.0000000

[[4]]

	variable	test	statistic	p.value
Kruskal-Wallis chi-squared	Self-medication	Kruskal-Wallis	42.12542	0.000001282789

[[5]]

	variable	test	comparison	Z	p.value
1	Self-medication	Dunn	GM of colour - Ind. men	0.6792212	1.000000000
2	Self-medication	Dunn	GM of colour - Ind. women	2.4930957	0.227942449
3	Self-medication	Dunn	Ind. men - Ind. women	2.4083570	0.288441061
4	Self-medication	Dunn	GM of colour - Men of colour	2.9204131	0.062922176
5	Self-medication	Dunn	Ind. men - Men of colour	2.8404680	0.081085300
6	Self-medication	Dunn	Ind. women - Men of colour	1.1181276	1.000000000
7	Self-medication	Dunn	GM of colour - TwoS	-0.4423907	1.000000000
8	Self-medication	Dunn	Ind. men - TwoS	-1.1619473	1.000000000
9	Self-medication	Dunn	Ind. women - TwoS	-2.9207676	0.062850628
**10**	**Self-medication**	**Dunn**	**Men of colour - TwoS**	**-3.2957972**	**0.017665711**
11	Self-medication	Dunn	GM of colour - White GM	2.1575970	0.557265264

	variable	test	comparison	Z	p.value
12	Self-medication	Dunn	Ind. men - White GM	1.8606031	1.000000000
13	Self-medication	Dunn	Ind. women - White GM	0.5569196	1.000000000
14	Self-medication	Dunn	Men of colour - White GM	-0.1405215	1.000000000
15	Self-medication	Dunn	TwoS - White GM	2.4992453	0.224024628
16	Self-medication	Dunn	GM of colour - White men	1.4859101	1.000000000
17	Self-medication	Dunn	Ind. men - White men	1.0090313	1.000000000
18	Self-medication	Dunn	Ind. women - White men	-1.8832328	1.000000000
19	Self-medication	Dunn	Men of colour - White men	-2.4055711	0.290649805
20	Self-medication	Dunn	TwoS - White men	1.9678958	0.883440418
21	Self-medication	Dunn	White GM - White men	-1.3908816	1.000000000
22	Self-medication	Dunn	GM of colour - White women	2.9122746	0.064585276
**23**	**Self-medication**	**Dunn**	**Ind. men - White women**	**3.1199375**	**0.032560098**
24	Self-medication	Dunn	Ind. women - White women	0.6423548	1.000000000
25	Self-medication	Dunn	Men of colour - White women	-0.8856994	1.000000000
**26**	**Self-medication**	**Dunn**	**TwoS - White women**	**3.3282099**	**0.015733080**
27	Self-medication	Dunn	White GM - White women	-0.3635915	1.000000000
**28**	**Self-medication**	**Dunn**	**White men - White women**	**3.0657180**	**0.039086644**
**29**	**Self-medication**	**Dunn**	**GM of colour - Women of colour**	**3.1611251**	**0.028288978**
**30**	**Self-medication**	**Dunn**	**Ind. men - Women of colour**	**3.4512263**	**0.010044817**
31	Self-medication	Dunn	Ind. women - Women of colour	1.2581224	1.000000000
32	Self-medication	Dunn	Men of colour - Women of colour	-0.4659232	1.000000000
**33**	**Self-medication**	**Dunn**	**TwoS - Women of colour**	**3.5590790**	**0.006698836**
34	Self-medication	Dunn	White GM - Women of colour	-0.1206129	1.000000000
**35**	**Self-medication**	**Dunn**	**White men - Women of colour**	**3.4973962**	**0.008456824**
36	Self-medication	Dunn	White women - Women of colour	1.1588240	1.000000000

[[6]]

	variable	test	statistic	p.value
Kruskal-Wallis chi-squared	Counselling	Kruskal-Wallis	111.2787	0.00000000000000000002078519

[[7]]

	variable	test	comparison	Z	p.value
1	Counselling	Dunn	GM of colour - Ind. men	-0.4885671	1.0000000000000000000
2	Counselling	Dunn	GM of colour - Ind. women	2.7216819	0.1169111166937845947
**3**	**Counselling**	**Dunn**	**Ind. men - Ind. women**	**4.5450978**	**0.0000988375436392490**
**4**	**Counselling**	**Dunn**	**GM of colour - Men of colour**	**3.3871435**	**0.0127123999227663920**
**5**	**Counselling**	**Dunn**	**Ind. men - Men of colour**	**4.8544745**	**0.0000217271542093317**
6	Counselling	Dunn	Ind. women - Men of colour	1.5313892	1.0000000000000000000
7	Counselling	Dunn	GM of colour - TwoS	0.3512413	1.0000000000000000000
8	Counselling	Dunn	Ind. men - TwoS	0.8589892	1.0000000000000000000
9	Counselling	Dunn	Ind. women - TwoS	-2.0377252	0.7483935940006540832
10	Counselling	Dunn	Men of colour - TwoS	-2.7585812	0.1044951747446033197
11	Counselling	Dunn	GM of colour - White GM	1.5619312	1.0000000000000000000
12	Counselling	Dunn	Ind. men - White GM	2.2018692	0.4981419043928149160
13	Counselling	Dunn	Ind. women - White GM	-0.3731416	1.0000000000000000000
14	Counselling	Dunn	Men of colour - White GM	-1.2277714	1.0000000000000000000
15	Counselling	Dunn	TwoS - White GM	1.1731729	1.0000000000000000000
**16**	**Counselling**	**Dunn**	**GM of colour - White men**	**3.5296754**	**0.0074892554110559265**
**17**	**Counselling**	**Dunn**	**Ind. men - White men**	**5.6192029**	**0.0000003453129004661**
18	Counselling	Dunn	Ind. women - White men	1.5852992	1.0000000000000000000
19	Counselling	Dunn	Men of colour - White men	-0.4253148	1.0000000000000000000
20	Counselling	Dunn	TwoS - White men	2.7826828	0.0970406636317809740
21	Counselling	Dunn	White GM - White men	1.0757371	1.0000000000000000000
**22**	**Counselling**	**Dunn**	**GM of colour - White women**	**4.6175828**	**0.0000698824524419083**
**23**	**Counselling**	**Dunn**	**Ind. men - White women**	**7.7119635**	**0.0000000000002230138**
**24**	**Counselling**	**Dunn**	**Ind. women - White women**	**4.2274617**	**0.0004254163325936895**
25	Counselling	Dunn	Men of colour - White women	0.6694675	1.0000000000000000000
**26**	**Counselling**	**Dunn**	**TwoS - White women**	**3.6999905**	**0.0038809355292426305**
27	Counselling	Dunn	White GM - White women	1.8234069	1.0000000000000000000
28	Counselling	Dunn	White men - White women	1.9378566	0.9475330559070068714
**29**	**Counselling**	**Dunn**	**GM of colour - Women of colour**	**4.6332336**	**0.0000647996235229681**
**30**	**Counselling**	**Dunn**	**Ind. men - Women of colour**	**7.6043206**	**0.0000000000005155280**
**31**	**Counselling**	**Dunn**	**Ind. women - Women of colour**	**4.0976481**	**0.0007512643089673778**
32	Counselling	Dunn	Men of colour - Women of colour	0.7577999	1.0000000000000000000
**33**	**Counselling**	**Dunn**	**TwoS - Women of colour**	**3.7294490**	**0.0034541803888970407**
34	Counselling	Dunn	White GM - Women of colour	1.8708157	1.0000000000000000000
35	Counselling	Dunn	White men - Women of colour	1.9792819	0.8601170327047091835
36	Counselling	Dunn	White women - Women of colour	0.3095971	1.0000000000000000000

[[8]]

	variable	test	statistic	p.value
Kruskal-Wallis chi-squared	Religion	Kruskal-Wallis	169.208	0.000000000000000000000000000000001890005

[[9]]

	variable	test	comparison	Z	p.value
1	Religion	Dunn	GM of colour - Ind. men	-0.5853676	1.00000000000000000000000000000000
2	Religion	Dunn	GM of colour - Ind. women	2.8079490	0.08974462500421569177699865349496
**3**	**Religion**	**Dunn**	**Ind. men - Ind. women**	**4.8158147**	**0.00002638809956476683029171284578**
4	Religion	Dunn	GM of colour - Men of colour	0.2802489	1.00000000000000000000000000000000
5	Religion	Dunn	Ind. men - Men of colour	1.0699015	1.00000000000000000000000000000000
**6**	**Religion**	**Dunn**	**Ind. women - Men of colour**	**-3.2658306**	**0.01964583669205237706489874938143**
7	Religion	Dunn	GM of colour - TwoS	-1.0648007	1.00000000000000000000000000000000
8	Religion	Dunn	Ind. men - TwoS	-0.6851859	1.00000000000000000000000000000000
**9**	**Religion**	**Dunn**	**Ind. women - TwoS**	**-3.9157222**	**0.00162241205574125075053459887897**
10	Religion	Dunn	Men of colour - TwoS	-1.4643493	1.00000000000000000000000000000000
11	Religion	Dunn	GM of colour - White GM	2.1793160	0.52754766805699571108334566815756
12	Religion	Dunn	Ind. men - White GM	2.9882013	0.05051243161242521817477779677574
13	Religion	Dunn	Ind. women - White GM	0.3171920	1.00000000000000000000000000000000
14	Religion	Dunn	Men of colour - White GM	2.1863816	0.51817856376296889120425248620450
**15**	**Religion**	**Dunn**	**TwoS - White GM**	**3.0540759**	**0.04063589378340297375569178939259**
16	Religion	Dunn	GM of colour - White men	1.9712179	0.87658116270424601967903299737372
17	Religion	Dunn	Ind. men - White men	3.6204343	0.00529396020581454022219114463610
18	Religion	Dunn	Ind. women - White men	-1.5601295	1.00000000000000000000000000000000
19	Religion	Dunn	Men of colour - White men	2.1582526	0.55634754455724577670849839705625
**20**	**Religion**	**Dunn**	**TwoS - White men**	**3.1458301**	**0.02981091696851387307098946166661**
21	Religion	Dunn	White GM - White men	-1.0075481	1.00000000000000000000000000000000
**22**	**Religion**	**Dunn**	**GM of colour - White women**	**3.0273994**	**0.04440020589849193871634369656931**
**23**	**Religion**	**Dunn**	**Ind. men - White women**	**5.5241627**	**0.00000059590900791728971608654519**
24	Religion	Dunn	Ind. women - White women	0.1043018	1.00000000000000000000000000000000
**25**	**Religion**	**Dunn**	**Men of colour - White women**	**3.7065084**	**0.00378245317208596122313113419011**
**26**	**Religion**	**Dunn**	**TwoS - White women**	**4.1563779**	**0.00058199919098502015269575027290**
27	Religion	Dunn	White GM - White women	-0.2957571	1.00000000000000000000000000000000
28	Religion	Dunn	White men - White women	2.1382427	0.58494689899257945597810248727910
29	Religion	Dunn	GM of colour - Women of colour	0.2562800	1.00000000000000000000000000000000
30	Religion	Dunn	Ind. men - Women of colour	1.3795196	1.00000000000000000000000000000000
**31**	**Religion**	**Dunn**	**Ind. women - Women of colour**	**-6.2061890**	**0.00000000977127930646765900830897**
32	Religion	Dunn	Men of colour - Women of colour	-0.1108023	1.00000000000000000000000000000000
33	Religion	Dunn	TwoS - Women of colour	1.6397268	1.00000000000000000000000000000000
34	Religion	Dunn	White GM - Women of colour	-2.5738357	0.18104042914662563101835246470728
**35**	**Religion**	**Dunn**	**White men - Women of colour**	**-4.0407634**	**0.00095899498966908096678207762409**
**36**	**Religion**	**Dunn**	**White women - Women of colour**	**-11.0402114**	**0.00000000000000000000000000440023**

[[10]]

	variable	test	statistic	p.value
Kruskal-Wallis chi-squared	Mindfullness	Kruskal-Wallis	39.79518	0.000003497777

[[11]]

	variable	test	comparison	Z	p.value
1	Mindfullness	Dunn	GM of colour - Ind. men	-1.388699270	1.0000000000
2	Mindfullness	Dunn	GM of colour - Ind. women	1.058219792	1.0000000000
**3**	**Mindfullness**	**Dunn**	**Ind. men - Ind. women**	**3.634316182**	**0.0050169400**
4	Mindfullness	Dunn	GM of colour - Men of colour	0.404081463	1.0000000000
5	Mindfullness	Dunn	Ind. men - Men of colour	2.226640396	0.4674837035
6	Mindfullness	Dunn	Ind. women - Men of colour	-0.793721539	1.0000000000
7	Mindfullness	Dunn	GM of colour - TwoS	-0.107916621	1.0000000000
8	Mindfullness	Dunn	Ind. men - TwoS	1.199346890	1.0000000000
9	Mindfullness	Dunn	Ind. women - TwoS	-1.136972540	1.0000000000
10	Mindfullness	Dunn	Men of colour - TwoS	-0.509362673	1.0000000000
11	Mindfullness	Dunn	GM of colour - White GM	-0.005162011	1.0000000000
12	Mindfullness	Dunn	Ind. men - White GM	1.205279520	1.0000000000
13	Mindfullness	Dunn	Ind. women - White GM	-0.902364504	1.0000000000
14	Mindfullness	Dunn	Men of colour - White GM	-0.359775261	1.0000000000
15	Mindfullness	Dunn	TwoS - White GM	0.093002090	1.0000000000
16	Mindfullness	Dunn	GM of colour - White men	1.139333639	1.0000000000

	variable	test	comparison	Z	p.value
**17**	**Mindfullness**	**Dunn**	**Ind. men - White men**	**3.705087568**	**0.0038037189**
18	Mindfullness	Dunn	Ind. women - White men	0.170345518	1.0000000000
19	Mindfullness	Dunn	Men of colour - White men	0.901022569	1.0000000000
20	Mindfullness	Dunn	TwoS - White men	1.213437128	1.0000000000
21	Mindfullness	Dunn	White GM - White men	0.973057335	1.0000000000
22	Mindfullness	Dunn	GM of colour - White women	1.347346144	1.0000000000
**23**	**Mindfullness**	**Dunn**	**Ind. men - White women**	**4.450790623**	**0.0001539985**
24	Mindfullness	Dunn	Ind. women - White women	0.545320946	1.0000000000
25	Mindfullness	Dunn	Men of colour - White women	1.201032819	1.0000000000
26	Mindfullness	Dunn	TwoS - White women	1.408351583	1.0000000000
27	Mindfullness	Dunn	White GM - White women	1.127036437	1.0000000000
28	Mindfullness	Dunn	White men - White women	0.296664825	1.0000000000
29	Mindfullness	Dunn	GM of colour - Women of colour	0.216488113	1.0000000000
30	Mindfullness	Dunn	Ind. men - Women of colour	2.700458836	0.1246390294
31	Mindfullness	Dunn	Ind. women - Women of colour	-2.061545587	0.7065183436
32	Mindfullness	Dunn	Men of colour - Women of colour	-0.370316557	1.0000000000
33	Mindfullness	Dunn	TwoS - Women of colour	0.347969387	1.0000000000
34	Mindfullness	Dunn	White GM - Women of colour	0.187340849	1.0000000000
35	Mindfullness	Dunn	White men - Women of colour	-2.183665416	0.5217631714
**36**	**Mindfullness**	**Dunn**	**White women - Women of colour**	**-4.491410748**	**0.0001273553**

[[12]]

	variable	test	statistic	p.value
Kruskal-Wallis chi-squared	Suport line	Kruskal-Wallis	149.3581	0.000000000000000000000000000002668763

[[13]]

	variable	test	comparison	Z	p.value
1	Suport line	Dunn	GM of colour - Ind. men	-0.60053107	1.000000000000000000000000
**2**	**Suport line**	**Dunn**	**GM of colour - Ind. women**	**4.19206514**	**0.000497567873449492070227**
**3**	**Suport line**	**Dunn**	**Ind. men - Ind. women**	**6.76385152**	**0.000000000241867429644044**
**4**	**Suport line**	**Dunn**	**GM of colour - Men of colour**	**3.59929359**	**0.005743490157194827000486**
**5**	**Suport line**	**Dunn**	**Ind. men - Men of colour**	**5.21532511**	**0.000003302912090464137830**
6	Suport line	Dunn	Ind. women - Men of colour	-0.01641108	1.000000000000000000000000
7	Suport line	Dunn	GM of colour - TwoS	0.20340408	1.000000000000000000000000
8	Suport line	Dunn	Ind. men - TwoS	0.80920431	1.000000000000000000000000
**9**	**Suport line**	**Dunn**	**Ind. women - TwoS**	**-3.69208885**	**0.004003555174024935742194**
**10**	**Suport line**	**Dunn**	**Men of colour - TwoS**	**-3.21550837**	**0.023438465505390193577284**
11	Suport line	Dunn	GM of colour - White GM	2.01106356	0.797737379564061654946272
12	Suport line	Dunn	Ind. men - White GM	2.81013906	0.089136177167998723036568
13	Suport line	Dunn	Ind. women - White GM	-1.06276061	1.000000000000000000000000
14	Suport line	Dunn	Men of colour - White GM	-0.94098990	1.000000000000000000000000
15	Suport line	Dunn	TwoS - White GM	1.77022630	1.000000000000000000000000
**16**	**Suport line**	**Dunn**	**GM of colour - White men**	**3.54457074**	**0.007078553444831270358151**
**17**	**Suport line**	**Dunn**	**Ind. men - White men**	**5.80657755**	**0.000000114772940590053354**
18	Suport line	Dunn	Ind. women - White men	-1.16122676	1.000000000000000000000000
19	Suport line	Dunn	Men of colour - White men	-0.75955836	1.000000000000000000000000
**20**	**Suport line**	**Dunn**	**TwoS - White men**	**3.08682329**	**0.036415408634166257506593**
21	Suport line	Dunn	White GM - White men	0.53839291	1.000000000000000000000000
**22**	**Suport line**	**Dunn**	**GM of colour - White women**	**5.40631644**	**0.000001158013692400001016**
**23**	**Suport line**	**Dunn**	**Ind. men - White women**	**9.07973819**	**0.000000000000000001959054**
24	Suport line	Dunn	Ind. women - White women	2.32915929	0.357311316764916342414438
25	Suport line	Dunn	Men of colour - White women	1.28787369	1.000000000000000000000000
**26**	**Suport line**	**Dunn**	**TwoS - White women**	**4.78660043**	**0.000030533413471244202160**
27	Suport line	Dunn	White GM - White women	1.89946159	1.000000000000000000000000
**28**	**Suport line**	**Dunn**	**White men - White women**	**3.70829116**	**0.003755927730110076179920**
**29**	**Suport line**	**Dunn**	**GM of colour - Women of colour**	**4.04032973**	**0.000960769896093571404948**
**30**	**Suport line**	**Dunn**	**Ind. men - Women of colour**	**6.92948868**	**0.000000000076025593671807**
31	Suport line	Dunn	Ind. women - Women of colour	-0.82028309	1.000000000000000000000000
32	Suport line	Dunn	Men of colour - Women of colour	-0.45336546	1.000000000000000000000000
**33**	**Suport line**	**Dunn**	**TwoS - Women of colour**	**3.51685760**	**0.007860383693148946526730**
34	Suport line	Dunn	White GM - Women of colour	0.80173748	1.000000000000000000000000
35	Suport line	Dunn	White men - Women of colour	0.63243558	1.000000000000000000000000
**36**	**Suport line**	**Dunn**	**White women - Women of colour**	**-5.19478802**	**0.000003689158383992510954**

[[14]]

	variable	test	statistic	p.value

Kruskal-Wallis chi-squared	Peer support	Kruskal-Wallis	22.28251	0.00441855

[[15]]

	variable	test	comparison	Z	p.value
1	Peer support	Dunn	GM of colour - Ind. men	-1.81952625	1.0000000000
2	Peer support	Dunn	GM of colour - Ind. women	0.36077919	1.0000000000
**3**	**Peer support**	**Dunn**	**Ind. men - Ind. women**	**3.33567087**	**0.0153168917**
4	Peer support	Dunn	GM of colour - Men of colour	-0.21869942	1.0000000000
5	Peer support	Dunn	Ind. men - Men of colour	1.95646739	0.9073821731
6	Peer support	Dunn	Ind. women - Men of colour	-0.80360263	1.0000000000
7	Peer support	Dunn	GM of colour - TwoS	-0.35187328	1.0000000000
8	Peer support	Dunn	Ind. men - TwoS	1.32687761	1.0000000000
9	Peer support	Dunn	Ind. women - TwoS	-0.79312338	1.0000000000
10	Peer support	Dunn	Men of colour - TwoS	-0.19083806	1.0000000000
11	Peer support	Dunn	GM of colour - White GM	0.78025378	1.0000000000
12	Peer support	Dunn	Ind. men - White GM	2.47396668	0.2405198875
13	Peer support	Dunn	Ind. women - White GM	0.65928927	1.0000000000
14	Peer support	Dunn	Men of colour - White GM	1.06261744	1.0000000000
15	Peer support	Dunn	TwoS - White GM	1.07809815	1.0000000000
16	Peer support	Dunn	GM of colour - White men	1.07880417	1.0000000000
**17**	**Peer support**	**Dunn**	**Ind. men - White men**	**4.28292107**	**0.0003320201**
18	Peer support	Dunn	Ind. women - White men	1.37857722	1.0000000000
19	Peer support	Dunn	Men of colour - White men	1.73488920	1.0000000000
20	Peer support	Dunn	TwoS - White men	1.46816149	1.0000000000
21	Peer support	Dunn	White GM - White men	-0.04419715	1.0000000000
22	Peer support	Dunn	GM of colour - White women	-0.12128554	1.0000000000
**23**	**Peer support**	**Dunn**	**Ind. men - White women**	**3.03417255**	**0.0434153576**
24	Peer support	Dunn	Ind. women - White women	-1.23771908	1.0000000000
25	Peer support	Dunn	Men of colour - White women	0.20089313	1.0000000000
26	Peer support	Dunn	TwoS - White women	0.36439321	1.0000000000
27	Peer support	Dunn	White GM - White women	-1.10805371	1.0000000000
28	Peer support	Dunn	White men - White women	-2.98050364	0.0517994668
29	Peer support	Dunn	GM of colour - Women of colour	-0.16206141	1.0000000000
30	Peer support	Dunn	Ind. men - Women of colour	2.88638075	0.0701460716
31	Peer support	Dunn	Ind. women - Women of colour	-1.24611152	1.0000000000
32	Peer support	Dunn	Men of colour - Women of colour	0.13813463	1.0000000000
33	Peer support	Dunn	TwoS - Women of colour	0.32042803	1.0000000000
34	Peer support	Dunn	White GM - Women of colour	-1.13327818	1.0000000000
35	Peer support	Dunn	White men - Women of colour	-2.87725962	0.0722061742
36	Peer support	Dunn	White women - Women of colour	-0.17086400	1.0000000000

[[16]]

	variable	test	statistic	p.value
Kruskal-Wallis chi-squared	Family	Kruskal-Wallis	29.53704	0.0002551303

[[17]]

	variable	test	comparison	Z	p.value
1	Family	Dunn	GM of colour - Ind. men	0.149210935	1.000000000
2	Family	Dunn	GM of colour - Ind. women	-0.096654738	1.000000000
3	Family	Dunn	Ind. men - Ind. women	-0.375183996	1.000000000
4	Family	Dunn	GM of colour - Men of colour	-1.276784813	1.000000000
5	Family	Dunn	Ind. men - Men of colour	-1.833177689	1.000000000
6	Family	Dunn	Ind. women - Men of colour	-1.844314352	1.000000000
7	Family	Dunn	GM of colour - TwoS	-0.104521585	1.000000000
8	Family	Dunn	Ind. men - TwoS	-0.264117469	1.000000000
9	Family	Dunn	Ind. women - TwoS	-0.043890121	1.000000000
10	Family	Dunn	Men of colour - TwoS	1.094326026	1.000000000
11	Family	Dunn	GM of colour - White GM	0.779180720	1.000000000
12	Family	Dunn	Ind. men - White GM	0.764701156	1.000000000
13	Family	Dunn	Ind. women - White GM	1.059741641	1.000000000
14	Family	Dunn	Men of colour - White GM	2.028049686	0.765993190
15	Family	Dunn	TwoS - White GM	0.851675667	1.000000000
16	Family	Dunn	GM of colour - White men	-0.008047968	1.000000000
17	Family	Dunn	Ind. men - White men	-0.245371527	1.000000000
18	Family	Dunn	Ind. women - White men	0.171855094	1.000000000
19	Family	Dunn	Men of colour - White men	1.941156107	0.940308215
20	Family	Dunn	TwoS - White men	0.126285906	1.000000000
21	Family	Dunn	White GM - White men	-0.977640314	1.000000000

```
22   Family Dunn      GM of colour - White women -1.107453620 1.000000000
23   Family Dunn          Ind. men - White women -1.965212648 0.889013352
24   Family Dunn        Ind. women - White women -2.522090386 0.209987511
25   Family Dunn     Men of colour - White women  0.617332872 1.000000000
26   Family Dunn              TwoS - White women -0.890406638 1.000000000
27   Family Dunn          White GM - White women -1.969922092 0.879251402
28   Family Dunn         White men - White women -2.635444988 0.151248681
29   Family Dunn  GM of colour - Women of colour -1.552184905 1.000000000
30   Family Dunn      Ind. men - Women of colour -2.600086479 0.167760486
31   Family Dunn    Ind. women - Women of colour -3.424713623 0.011078060
32   Family Dunn Men of colour - Women of colour -0.052882137 1.000000000
33   Family Dunn          TwoS - Women of colour -1.308775663 1.000000000
34   Family Dunn      White GM - Women of colour -2.346620809 0.341001425
35   Family Dunn     White men - Women of colour -3.502916232 0.008283495
36   Family Dunn   White women - Women of colour -1.907309578 1.000000000

[[18]]
                                     variable           test statistic             p.value
Kruskal-Wallis chi-squared Supervisor support Kruskal-Wallis   66.4156 0.00000000002531586

[[19]]
             variable test                     comparison           Z             p.value
1  Supervisor support Dunn         GM of colour - Ind. men  0.51006723 1.0000000000000000
2  Supervisor support Dunn       GM of colour - Ind. women  3.11582105 0.0330180391776542
3  Supervisor support Dunn           Ind. men - Ind. women  3.53607795 0.0073100671985604
4  Supervisor support Dunn    GM of colour - Men of colour  1.44310026 1.0000000000000000
5  Supervisor support Dunn        Ind. men - Men of colour  1.18752747 1.0000000000000000
6  Supervisor support Dunn      Ind. women - Men of colour -1.91762387 0.9928569743370631
7  Supervisor support Dunn             GM of colour - TwoS  0.69228926 1.0000000000000000
8  Supervisor support Dunn                 Ind. men - TwoS  0.32524294 1.0000000000000000
9  Supervisor support Dunn               Ind. women - TwoS -1.96485618 0.8897559395594488
10 Supervisor support Dunn            Men of colour - TwoS -0.56209303 1.0000000000000000
11 Supervisor support Dunn         GM of colour - White GM  0.60615505 1.0000000000000000
12 Supervisor support Dunn             Ind. men - White GM  0.24428777 1.0000000000000000
13 Supervisor support Dunn           Ind. women - White GM -1.88943957 1.0000000000000000
14 Supervisor support Dunn        Men of colour - White GM -0.58928613 1.0000000000000000
15 Supervisor support Dunn                 TwoS - White GM -0.05145818 1.0000000000000000
16 Supervisor support Dunn        GM of colour - White men  1.74847592 1.0000000000000000
17 Supervisor support Dunn            Ind. men - White men  1.63115911 1.0000000000000000
18 Supervisor support Dunn          Ind. women - White men -2.56855487 0.1838223791893637
19 Supervisor support Dunn       Men of colour - White men  0.13667646 1.0000000000000000
20 Supervisor support Dunn                TwoS - White men  0.72366360 1.0000000000000000
21 Supervisor support Dunn            White GM - White men  0.73811502 1.0000000000000000
22 Supervisor support Dunn      GM of colour - White women  2.71667152 0.1186956321850177
23 Supervisor support Dunn          Ind. men - White women  3.12851668 0.0316243874017827
24 Supervisor support Dunn        Ind. women - White women -1.45885983 1.0000000000000000
25 Supervisor support Dunn     Men of colour - White women  1.31948751 1.0000000000000000
26 Supervisor support Dunn              TwoS - White women  1.52787003 1.0000000000000000
27 Supervisor support Dunn          White GM - White women  1.47651833 1.0000000000000000
28 Supervisor support Dunn         White men - White women  1.96991208 0.8792720681013702
29 Supervisor support Dunn  GM of colour - Women of colour  1.03728787 1.0000000000000000
30 Supervisor support Dunn      Ind. men - Women of colour  0.63947907 1.0000000000000000
31 Supervisor support Dunn    Ind. women - Women of colour -5.13183295 0.0000051648140736
32 Supervisor support Dunn Men of colour - Women of colour -0.95545678 1.0000000000000000
33 Supervisor support Dunn          TwoS - Women of colour  0.02367267 1.0000000000000000
34 Supervisor support Dunn      White GM - Women of colour  0.09068035 1.0000000000000000
35 Supervisor support Dunn     White men - Women of colour -1.79202858 1.0000000000000000
36 Supervisor support Dunn   White women - Women of colour -6.63259718 0.0000000005936959

[[20]]
                           variable           test statistic    p.value
Kruskal-Wallis chi-squared Outdoors Kruskal-Wallis  19.83819 0.01096589

[[21]]
   variable test                     comparison           Z    p.value
1  Outdoors Dunn         GM of colour - Ind. men  1.47549161 1.00000000
2  Outdoors Dunn       GM of colour - Ind. women  1.74355025 1.00000000
3  Outdoors Dunn           Ind. men - Ind. women  0.12732425 1.00000000
```

4	Outdoors	Dunn	GM of colour - Men of colour	1.19483399	1.00000000
5	Outdoors	Dunn	Ind. men - Men of colour	-0.30824450	1.00000000
6	Outdoors	Dunn	Ind. women - Men of colour	-0.48979760	1.00000000
7	Outdoors	Dunn	GM of colour - TwoS	0.58818086	1.00000000
8	Outdoors	Dunn	Ind. men - TwoS	-0.69135987	1.00000000
9	Outdoors	Dunn	Ind. women - TwoS	-0.84449706	1.00000000
10	Outdoors	Dunn	Men of colour - TwoS	-0.44845219	1.00000000
11	Outdoors	Dunn	GM of colour - White GM	1.80865979	1.00000000
12	Outdoors	Dunn	Ind. men - White GM	0.76943072	1.00000000
13	Outdoors	Dunn	Ind. women - White GM	0.75884072	1.00000000
14	Outdoors	Dunn	Men of colour - White GM	0.97051222	1.00000000
15	Outdoors	Dunn	TwoS - White GM	1.19310482	1.00000000
16	Outdoors	Dunn	GM of colour - White men	1.25575656	1.00000000
17	Outdoors	Dunn	Ind. men - White men	-0.53129566	1.00000000
18	Outdoors	Dunn	Ind. women - White men	-0.90801187	1.00000000
19	Outdoors	Dunn	Men of colour - White men	-0.13756969	1.00000000
20	Outdoors	Dunn	TwoS - White men	0.40486040	1.00000000
21	Outdoors	Dunn	White GM - White men	-1.15877830	1.00000000
22	Outdoors	Dunn	GM of colour - White women	0.78980486	1.00000000
23	Outdoors	Dunn	Ind. men - White women	-1.43481338	1.00000000
24	Outdoors	Dunn	Ind. women - White women	-2.59960221	0.16799739
25	Outdoors	Dunn	Men of colour - White women	-0.92198386	1.00000000
26	Outdoors	Dunn	TwoS - White women	-0.07284285	1.00000000
27	Outdoors	Dunn	White GM - White women	-1.67798199	1.00000000
28	Outdoors	Dunn	White men - White women	-1.29600588	1.00000000
29	Outdoors	Dunn	GM of colour - Women of colour	1.59480313	1.00000000
30	Outdoors	Dunn	Ind. men - Women of colour	-0.20315113	1.00000000
31	Outdoors	Dunn	Ind. women - Women of colour	-0.54028891	1.00000000
32	Outdoors	Dunn	Men of colour - Women of colour	0.21319461	1.00000000
33	Outdoors	Dunn	TwoS - Women of colour	0.66602282	1.00000000
34	Outdoors	Dunn	White GM - Women of colour	-0.98172499	1.00000000
35	Outdoors	Dunn	White men - Women of colour	0.59102200	1.00000000
**36**	**Outdoors**	**Dunn**	**White women - Women of colour**	**3.28444155**	**0.01839324**
